# Anti-CMV therapy, what next? A systematic review

**DOI:** 10.3389/fmicb.2023.1321116

**Published:** 2023-11-20

**Authors:** Claire Gourin, Sophie Alain, Sébastien Hantz

**Affiliations:** ^1^INSERM, CHU Limoges, University of Limoges, RESINFIT, Limoges, France; ^2^CHU Limoges, Laboratoire de Bactériologie-Virologie-Hygiène, National Reference Center for Herpesviruses, Limoges, France

**Keywords:** cytomegalovirus, letermovir, maribavir, direct antivirals, indirect antivirals, immunomodulatory molecules, immunoglobulins

## Abstract

Human cytomegalovirus (HCMV) is one of the main causes of serious complications in immunocompromised patients and after congenital infection. There are currently drugs available to treat HCMV infection, targeting viral polymerase, whose use is complicated by toxicity and the emergence of resistance. Maribavir and letermovir are the latest antivirals to have been developed with other targets. The approval of letermovir represents an important innovation for CMV prevention in hematopoietic stem cell transplant recipients, whereas maribavir allowed improving the management of refractory or resistant infections in transplant recipients. However, in case of multidrug resistance or for the prevention and treatment of congenital CMV infection, finding new antivirals or molecules able to inhibit CMV replication with the lowest toxicity remains a critical need. This review presents a range of molecules known to be effective against HCMV. Molecules with a direct action against HCMV include brincidofovir, cyclopropavir and anti-terminase benzimidazole analogs. Artemisinin derivatives, quercetin and baicalein, and anti-cyclooxygenase-2 are derived from natural molecules and are generally used for different indications. Although they have demonstrated indirect anti-CMV activity, few clinical studies were performed with these compounds. Immunomodulating molecules such as leflunomide and everolimus have also demonstrated indirect antiviral activity against HCMV and could be an interesting complement to antiviral therapy. The efficacy of anti-CMV immunoglobulins are discussed in CMV congenital infection and in association with direct antiviral therapy in heart transplanted patients. All molecules are described, with their mode of action against HCMV, preclinical tests, clinical studies and possible resistance. All these molecules have shown anti-HCMV potential as monotherapy or in combination with others. These new approaches could be interesting to validate in clinical trials.

## 1 Introduction

Human cytomegalovirus (CMV) is an opportunistic pathogen in the immunocompromised host. Not only in transplant recipients, but also in AIDS patients or highly immunocompromised patients with congenital immunodeficiency or immunosuppressive biotherapies. Such infections can lead to graft rejection and organ damages (Kotton et al., 2018; Ljungman et al., 2019). Due to the use of preventive strategies, either preemptive treatment or prophylaxis, CMV disease frequency has decreased. But in solid organ recipients, late disease may occur in up to 18% of patients after stopping prophylaxis (Kotton et al., 2018). In stem cell recipients, it decreased from 10–40% to 2–3% in randomized trials but 5–10% in real life cohorts despite efficient preemptive treatment (Ljungman et al., 2019). Currently, available antivirals are limited to virostatic polymerase inhibitors (ganciclovir, its oral prodrug valganciclovir, cidofovir and foscarnet). Neutropenia limits efficacy of ganciclovir or valganciclovir and this hematological toxicity prevents its use as a prophylaxis in the stem cell recipients. Cidofovir and foscarnet are highly nephrotoxic and restricted to second line treatment. The second limitation of these molecules is the emergence of resistance, favored by prolonged treatments in highly immunocompromised hosts, and use of lower doses due to renal impairment (Razonable et al., 2019).

Congenital CMV infection (cCMV) is also a leading cause of hearing loss and neurological sequelae in children. During pregnancy, the prevalence of primary CMV infection ranges from 1 to 2% in the United States and Western Europe (Hyde et al., 2010; Leruez-Ville et al., 2020), with an average cCMV birth prevalence of 0.65% (Kenneson and Cannon, 2007). If the primary maternal infection occurs during pregnancy, especially during the first trimester, more severe sequelae, including complete hearing loss, are to be feared. The risk of maternal transmission occurs in 30–40% of case with CMV primary infection. Thus, during the first trimester of pregnancy, it is essential to prevent viral transmission to the fetus to avoid neurological disability in newborns (Ornoy and Diav-Citrin, 2006; Ross et al., 2006; Chatzakis et al., 2020). Among infected neonates, 12.7% will have symptoms at birth and 40 to 58% develop permanent sequelae. As a whole, long-term sequelae from sensorineural hearing loss to neurodevelopmental disabilities may occur in 17 to 19% of infected newborns, 51 to 57% of them following maternal primary infection (Dollard et al., 2007; Leruez-Ville and Ville, 2020). Ganciclovir (GCV) and its prodrug valganciclovir (VGCV), foscarnet (FOS) and cidofovir (CDV), are proscribed during pregnancy, due to their toxicity (e.g., neutropenia, nephrotoxicity). Although a randomized study has demonstrated the efficacy of a high dose (8 g per day) of valaciclovir (VACV), a prodrug of acyclovir, in preventing transmission, only 50% of periconceptional or 1st trimester primary infection transmissions were avoided, and more efficient anti-CMV drugs are thus needed (Shahar-Nissan et al., 2020). Treatment of symptomatic newborns for 6 weeks GCV or 6 months with VGCV was shown to improve hearing skills, and is now recommended, although 49 to 63% of the treated neonates developed grade 3 or 4 neutropenia with treatment (Kimberlin et al., 2015).

The burden of long-term therapies for immunocompromised patients, and the emergence of new resistance mechanisms (Chou, 2020), the unmet need for low toxic treatments to prevent or cure cCMV, make it essential to find new antiviral targets and to develop new therapies, in order to treat CMV infections more efficiently while reducing side effects.

Recently, two antiviral drugs with new targets, high specificity and low toxicity, reached clinical development: letermovir targets the highly virus-specific terminase complex (UL56, UL98 and UL51) and maribavir inhibits the UL97 viral kinase. Letermovir (LMV) was approved in 2017 by the Food and Drug Administration (FDA) for the prophylaxis of CMV infection in hematopoietic stem cell transplant patients with high risk of CMV infections (Marty et al., 2017). This new antiviral inhibits the terminase complex, a viral component not found in human cells, thereby reducing its toxicity. Similarly, maribavir (MBV) was approved in 2021 for the treatment of adults and children presenting post-transplant CMV infections refractory or resistant to antivirals (Food and Drug Administration, 2021). It targets the viral kinase UL97 (Biron et al., 2002). Both LMV and MBV have a high oral bioavailability and a low toxicity profile. Nevertheless, resistance mutations have already been described with these new antivirals, making it crucial to continue to develop new therapies.

**Figure 1:**
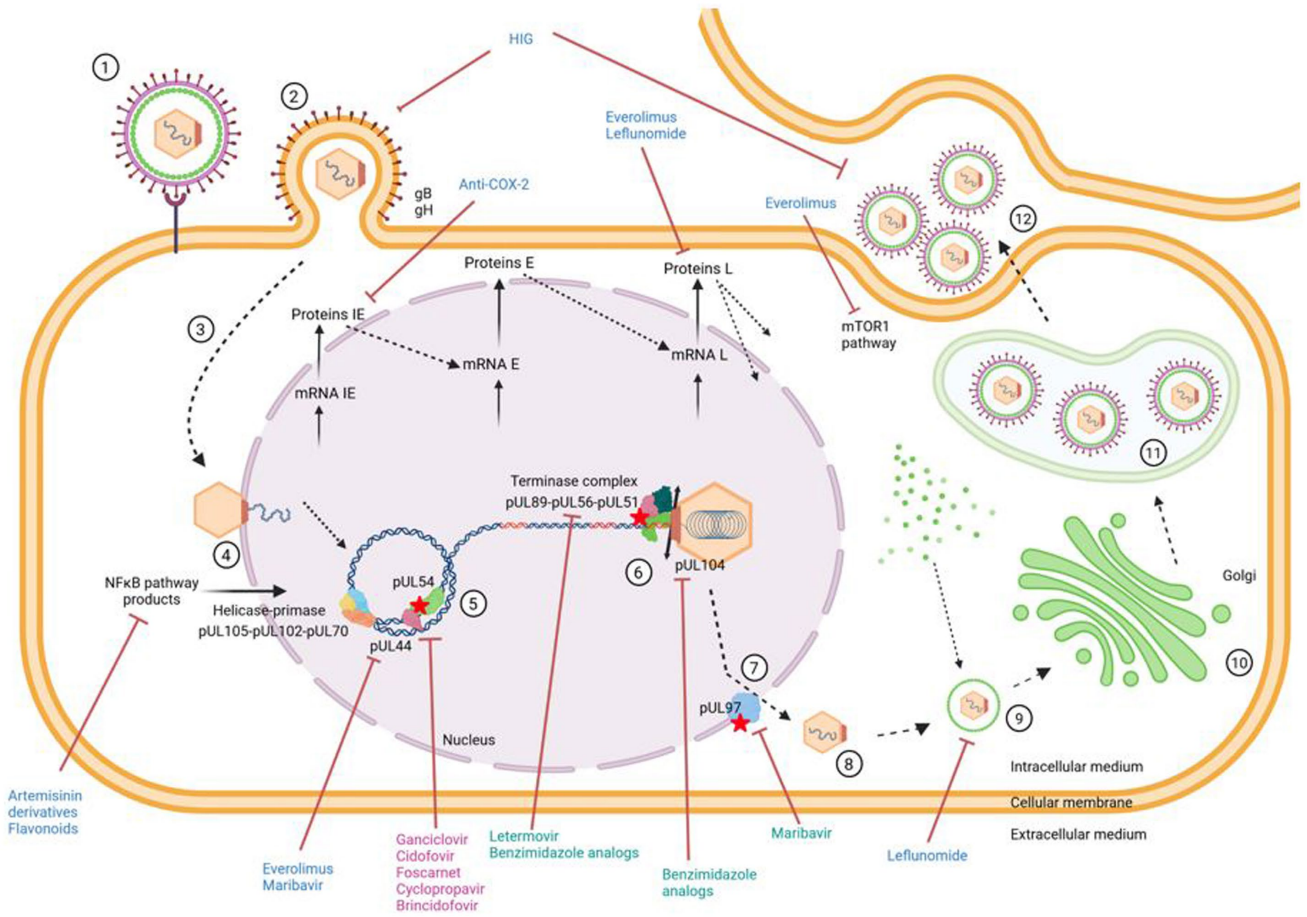
Antiviral targets in relication cycle of CMV. ① Virus attaches to cell. ② Entry of virus into cell and release of capsid into cytoplasm. ③ Migration of capsid to cell nucleus. ④ Release of viral genome from capsid through nuclear pore. ⑤ Replication of viral DNA by the rolling circle method using the viral polymerase pUL54. ⑥ Encapsidation of the genome into neoformed capsids via the encapsidation complex. ⑦ Nuclear exit. ⑧ Release of newly formed virions into cell cytoplasm. ⑨ Tegumentation of newly formed virions. ➉ Passage of virions through Golgi apparatus. ⑪ Acquisition of a primary envelope and transport of virions in a vesicle to the extracellular medium. ⑫ Budding, release of infectious virus particles and infection of a new cell. Indirect antivirals, direct antivirals and antivirals targeting the viral polymerase are in blue, green and pink, respectively. Created with BioRender.com

**Figure 2:**
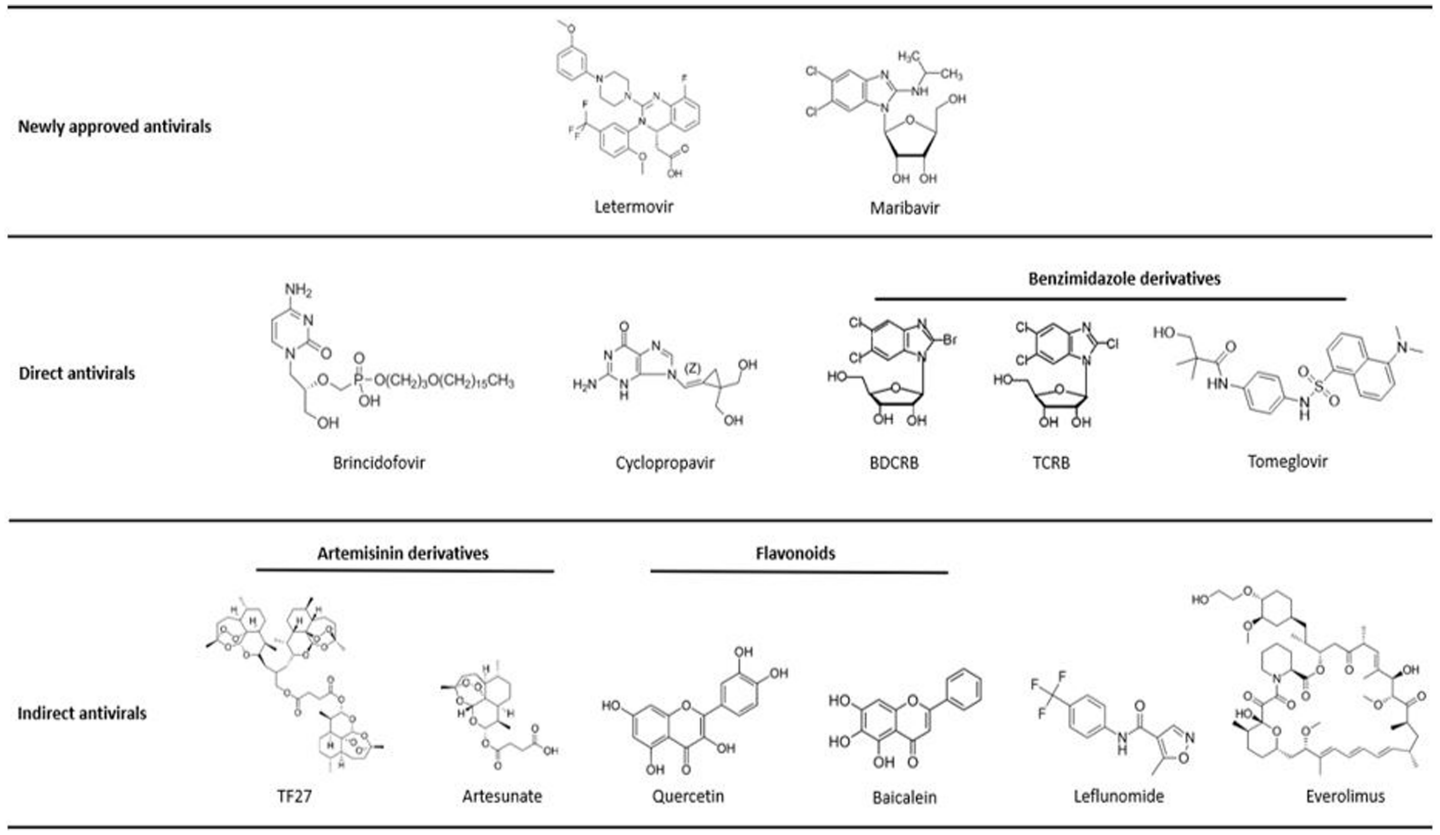
Molecule structures. Structures of chemical molecules with their corresponding order in the review. All molecules are classified as approved antivirals, molecules with direct antiviral activity or molecules with indirect antiviral activity.

This is why it is necessary to find new molecules with an anti-CMV spectrum. In this context, this review summarizes the panel of molecules with antiviral activity, including direct inhibitors (brincidofovir, cyclopropavir, anti-terminase benzimidazole analogs), molecules acting through cellular pathways inhibition (artemisinin derivatives, flavonoids, leflunomide, everolimus, or anti Cox) and immunoglobulins (Figures 1, 2 and Table 1).

**Table 1:** Summary of molecules and their main characteristics.

Molecule	Preclinical testing		Clinical trials				EC50	EC90	Selectivity index (EC50/CC50)	
	*In vitro*	*Ex vivo*	Animal model	Phase I	Phase II	Phase III				
Letermovir	Lischka et al. (2010) and Marschall et al. (2012)	-	Mouse xenograft (Lischka et al., 2010)		Chemaly et al. (2014), Stoelben et al. (2014), and Lischka et al. (2016)	NCT02137772 (Marty et al., 2017)	0.0038 ± 0.0009 μM (Lischka et al., 2010)	0.0051 ± 0.0014 μM (Lischka et al., 2010)	>18,000 (median SI) (Lischka et al., 2010)	
Maribavir	Lu and Thomas (2000) and Chou et al. (2012a)	-	Mouse, rats, monkeys, guinea pig, rabbit, dog (Koszalka et al., 2002)	Wang et al. (2003), Swan et al. (2007), and Winston et al. (2008)	Papanicolaou et al. (2019), Maertens et al. (2020), and Song et al. (2023)	NCT02931539 (Avery et al., 2021)	0.54 ± 0.06 μM (Biron et al., 2002) 19.4 ± 18.6 (Williams et al., 2003)	-	13 (Williams et al., 2003)	
Brincidofovir	Beadle et al. (2002)	-	Guinea pig Bravo et al., 2011 Monkeys, mice, rabbits, rats, cynomolgous monkeys	Painter et al. (2012)	NCT00942305 (Marty et al., 2013) NCT00942305 (Lanier et al., 2016)	NCT01769170 (Marty et al., 2019)	0.0009 μM (Beadle et al., 2002) 0.001 ± 0.001 μM (Williams-Aziz et al., 2005)	-	1 × 10^5^ (Beadle et al., 2002)	
Cyclopropavir	Kern et al. (2004a), Komazin-Meredith et al. (2014), Zhou et al. (2004), Kern et al. (2005), and Brooks and Bowlin (2013)	-	SCID mice (Bidanset et al., 2004; Kern et al., 2004a) dog, rat (Brooks and Bowlin, 2013)	(NTC01433835) (Brooks et al., 2015) (NCT02454699) (Rouphael et al., 2019)	-	-	1.2 ± 0.8 μM (Kern et al., 2005) 0.27–0.49 μM (Zhou et al., 2004)	-	1 × 10^5^ (Beadle et al., 2002)	
Flavonoids: Quercetin Baicalein	Cotin et al. (2012)	-	-	Polansky et al. (2016) and Polansky et al. (2018)	-	-	4.8 ± 1.2 (Cotin et al., 2012)	-	1.3 (Cotin et al., 2012)	
	Cotin et al. (2012)	-	Rats, mouse (Lai et al., 2003; Dou et al., 2011; Tian et al., 2012)	Li et al. (2014) and Li et al. (2021)	-	-	2.2 ± 0.5 (Cotin et al., 2012)	-	3 (Cotin et al., 2012)	
Anti-COX-2	Cotin et al. (2012), Andouard et al. (2021), and Baryawno et al. (2011)	Baryawno et al. (2011)	Mice (Baryawno et al., 2011)				8,6–22,1 ± 3,6–10,1 μM (Andouard et al., 2021)	-	3–10 (Andouard et al., 2021)	
Artemisinin derivatives: Artesunate TF27	Hutterer et al. (2015)	-	mice, rats and dogs (Efferth et al., 2002; Kaptein et al., 2006)	Shapira et al. (2008) and Germi et al. (2014)	-	-	18.5 ± 5.2 μM (Arav-Boger et al., 2010) 3.9 ± 0.6 μM (Hutterer et al., 2015)	-	4 ± 2 (Arav-Boger et al., 2010)	
	Hutterer et al. (2015)	Placental vili (Jacquet et al., 2020)	immunodefective mouse strain Rag−/− (Sonntag et al., 2019)	-	-	-	0.04 ± 0.01 μM (Reiter et al., 2015) 0.04 ± 0.01 μM (Hutterer et al., 2015)	0.08 ± 0.03 μM (Hutterer et al., 2015)	-	
Benzimidazole analogs: BDCRB TRCB Tomeglovir	Townsend et al. (1995)	-	Guinea pig (Nixon and McVoy, 2004; Kern et al., 2004a; Ourahmane et al., 2018)	-	-	-	0.7 μM (Townsend et al., 1995) 0.31 ± 0.06 (Biron et al., 2002) 2.7 ± 0.8 (Evers et al., 2002) 0.4 ± 0.3 μM (Williams et al., 2003)	0.9 ± 0.2 (Evers et al., 2002)	425 (Williams et al., 2003)	
	Townsend et al. (1995)	-		-	-	-	1.4 (Migawa et al., 1998) 2.9 μM (Townsend et al., 1995)	1.4 μM (Townsend et al., 1995)	-	
	Evers et al. (2002), Reefschlaeger et al. (2001), and McSharry et al. (2001)	-	Guinea pigs (Schleiss et al., 2005) SCID mice (Reefschlaeger et al., 2001; Weber et al., 2001)	-	-	-	0.52 ± 0.14 μM (Reefschlaeger et al., 2001)	-	300 (Reefschlaeger et al., 2001)	
Leflunomide	Waldman et al. (1999a)	-	Nude rats (Waldman et al., 1999a) Nude rats allograft (Chong et al., 2006)	Williams et al. (2002) and John et al. (2005)	-	-	40-60 μM (Waldman et al., 1999a)	-	-	
Everolimus										
Immunoglobulins	Coste Mazeau et al. (2022), Germer et al. (2016), Miescher et al. (2015), and Schampera et al. (2017)	Placental vili (Coste Mazeau et al., 2022)	Guinea pig (Bia et al., 1980; Bratcher et al., 1995; Chatterjee et al., 2001; Schleiss, 2008) Mouse (Cekinović et al., 2008)	Alsuliman et al. (2018)	NCT00881517 (Revello et al., 2014; Chiaie et al., 2018)	NCT01376778 (Hughes et al., 2021)	0.024 μM (Coste Mazeau et al., 2022)	-	-	

## 2 Newly approved antivirals target the late stage of the viral cycle

### 2.1 Letermovir

Letermovir (LMV; AIC246; Prevymis™; Figure 2), an antiviral of the quinazoline class, was developed by Aicuris and further marketed by Merck. LMV acts at the late stage of the viral cycle by direct inhibition of the human CMV terminase complex (Goldner et al., 2011). This viral terminase complex has no functional equivalent in the mammalian cells and the drug is therefore highly specific.

It appears to be very specific of CMV, and has a high activity against resistant strains to DNA polymerase inhibitors (Lischka et al., 2010). In 2017, LMV has been approved by the FDA for CMV prophylaxis in stem cell transplant patients seropositive for CMV (Marty et al., 2017).

#### 2.1.1 Mechanism of action

LMV targets pUL56, the large subunit of the CMV terminase complex that cleaves DNA prior to encapsidation of the genome in neoformed capsids. In addition, it has high specificity against CMV, even if other herpesviruses also possess a terminase complex. This could possibly be explained by a particular mode of action, as LMV probably disrupts the interaction between the subunits of the terminase complex: pUL56, pUL89 and pUL51 unlike other antivirals, which often act by blocking functional domains. LMV has been shown to inhibit primarily the viral step of genome encapsidation (Lischka et al., 2010). Moreover, it was demonstrated that LMV prevents cleavage of concatemeric DNA into units of genomes and formation of CMV mature virions (Goldner et al., 2011).

#### 2.1.2 Preclinical studies

Preclinical studies showed its very high antiviral activity (range: 1.6–5.1 nM; 1,000-fold more potent than GCV) against clinical and laboratory strains included refractory-resistant isolates to current drugs (Marschall et al., 2012) with low toxicity levels at high doses over the EC_90_. LMV has a high specificity to HCMV and is well tolerated in various cell types with a mean selectivity index of 18,000. LMV has *in vivo* efficacy in a mouse xenograft model (Lischka et al., 2010) and shows an anti-CMV activity in histoculture of third trimester placenta (Hamilton et al., 2020). It reached concentrations above EC_50_ at the fetal face when perfused across a third trimester placenta (Faure Bardon et al., 2020). However, its efficacy during the first trimester is not yet validated. Drug combination assays showed additive effect and no synergistic toxicity with current CMV drugs and no effect with anti-HIV drugs (Wildum et al., 2015).

#### 2.1.3 Clinical studies

LMV is a highly lipophilic molecule with a C_max_ of between 45 min and 2.25 h and a half-life of 12 h. After administration, LMV is highly protein-bound and eliminated via the biliary tract. The efficacy, safety and pharmacokinetic parameters of oral LMV were studied in a Phase IIa trial: LMV 40 mg twice daily or 80 mg once daily was administered to patients for 14 days as a preventive treatment against CMV infection in kidney and kidney/pancreas transplant recipients (Stoelben et al., 2014). This study demonstrated that all patients responded to LMV treatment. Chemaly et al. conducted a Phase IIb variable-dose prophylaxis trial in 2014: LMV was administered daily orally at 60 mg, 120 mg or 240 mg for 12 weeks post-transplant in CMV-seropositive allogeneic hematopoietic cell recipients. The incidence of prophylaxis failure (with or without virological failure) was significantly lower in the LMV-treated groups than in the placebo group (32% for the 120 mg group, 29% for the 240 mg group vs. 64%). The incidence of virological failure was lower in the 240 mg group (6%) than in the placebo group (36%). This study demonstrated that LMV was well tolerated and that a dose of 240 mg once daily was effective in suppressing viremia (Chemaly et al., 2014).

The Phase III prophylaxis trial (NCT02137772) evaluated the efficacy of a daily oral or intravenous dose of LMV of 480 mg/day (or 240 mg/day in patients taking ciclosporin) for 14 weeks after transplantation. A significant reduction in the number of patients developing CMV infection was observed. Indeed, at week 24, 38% of patients in the LMV group developed an HCMV infection versus 61% in the placebo group. In addition, the mortality rate was higher in the placebo group (16%) compared to the LMV group (10%). This study confirms the efficacy of LMV in the prophylaxis of CMV infection after HSCT in R+ patients (Marty et al., 2017). Used as primary and secondary prophylaxis in the French Compassionate Use Program (CUP) for high-risk patients, it was well tolerated and reduced the number of CMV infections compared with historical studies (Robin et al., 2020; Beauvais et al., 2022).

LMV has also been tested in case-series for prophylaxis or treatment in organ transplanted patients. It had good virologic outcomes and was well tolerated in patient with few side effects (Aryal et al., 2019; Veit et al., 2020; Linder et al., 2021). Recently, a large study conducted in 94 centers with kidney recipients showed that LMV was non-inferior to valganciclovir for prophylaxis of CMV disease for 52 weeks, with lower rates of leukopenia or neutropenia, arguing in favor of its use in this indication (Limaye et al., 2023).

#### 2.1.4 Resistance

**Figure 3:**
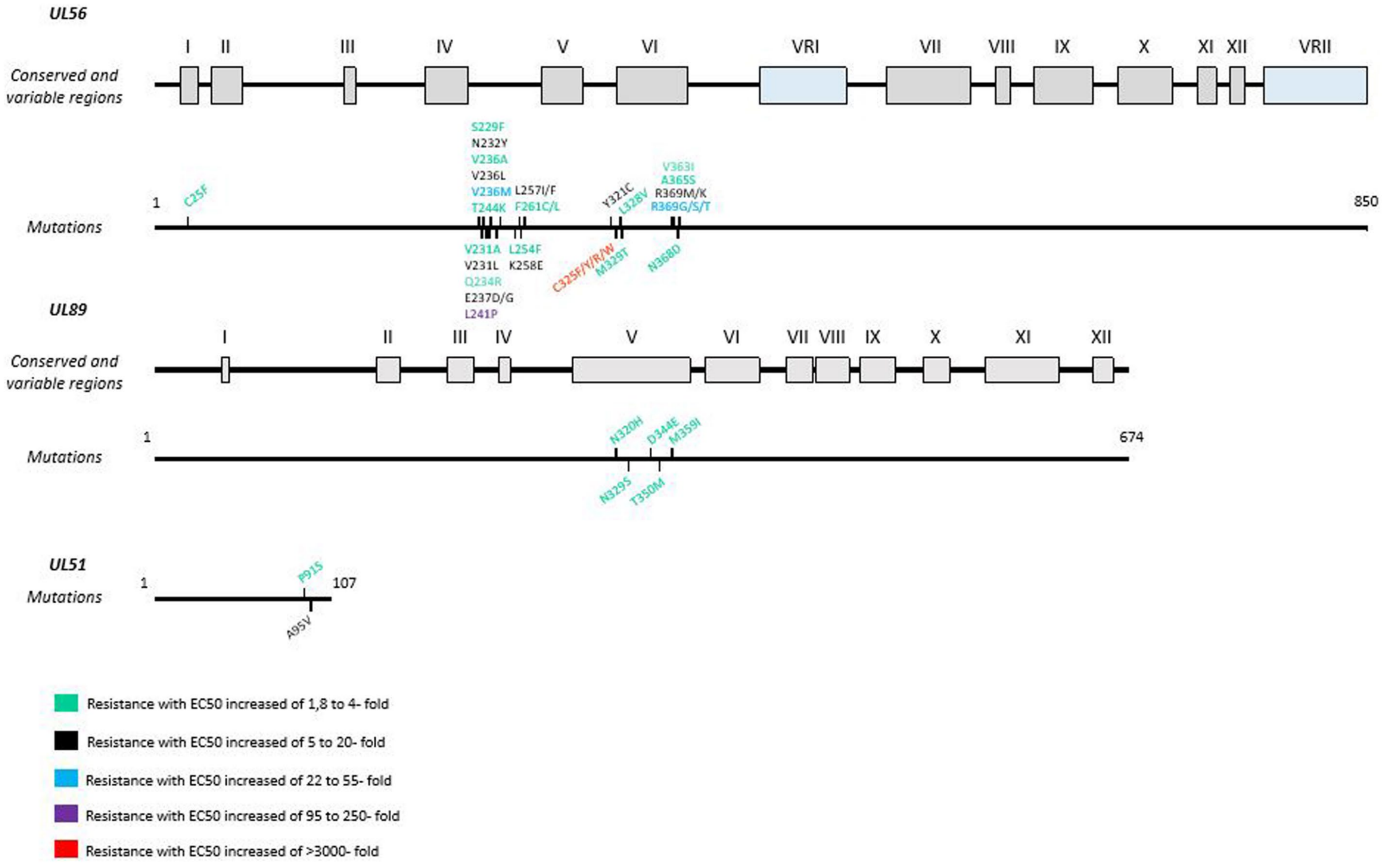
Resistance mutations to letermovir in *UL56*, *UL89* and *UL51* genes. LMV resistance mutations according to EC50 values. Mutations are referenced on genes under the map of conserved and variable regions. Scaled representation.

In less than 5 passages, the selection of resistant strains is rapidly achieved *in vitro* with UL56 mutations conferring high or absolute LMV resistance (Chou, 2017). *In vitro* studies have also revealed mutations in the genes encoding pUL51 and pUL89. In clinical trials, the first resistant isolate appeared after a sub-optimal dose of 60 mg/day (Chemaly et al., 2014; Lischka et al., 2016). Resistant mutants can emerge rapidly under LMV if treatment is interrupted or underdosed (Alain et al., 2020). pUL56 is the main target of LMV, which explains why mutations occur more frequently in this protein than in other proteins of the terminize complex (Cherrier et al., 2018; Frietsch et al., 2019; Alain et al., 2020). A new resistance mutation A95V in pUL51 was also described *in vivo* after LMV treatment in combination with a L257I mutation in pUL56 (Muller et al., 2022; Figure 3).

### 2.2 Maribavir

Maribavir (MBV; 1,263 W94; LIVTENCITY™) [formerly 1,263 W94, 5,6-dichloro-2-(isopropylamino)-1,β-l-ribofuranosyl-1-H-benzimidazole] (Figure 2) is an oral bioavailable benzimidazole riboside initially developed by Glaxo Smith Kline (Biron et al., 2002), then Viropharma, now marketed by Takeda Pharmaceuticals/a Shire company for treatment of refractory or resistant CMV infections. In November 2021, the FDA approved MBV for 400 mg twice a day-treatment of adults and children (12 years of age or older, weight > 35 kg) with post-transplant CMV infection/illness refractory/resistant to GCV, VGCV, CDV and FOS (Food and Drug Administration, 2021; Halpern-Cohen and Blumberg, 2022). In 2022, the European Commission approved MBV in the same indications.

#### 2.2.1 Mechanism of action

Maribavir does not require activation or intracellular processing. Unlike other anti-CMV drugs, MBV targets the viral kinase *UL97* and its natural substrates, which are involved in the DNA replication and viral capsid nuclear egress (Biron et al., 2002; Hamirally et al., 2009; Prichard, 2009). This mechanism of action confers MBV an *in vitro* and *in vivo* activity against GCV, FOS and CDV resistant CMV strains (Biron et al., 2002; Drew et al., 2006). The combination of MBV and GCV is therefore not recommended, as GCV activation requires three phosphorylation, the first of which being mediated by pUL97. Indeed, MBV antagonizes anti-CMV effect of GCV by increasing the 50% inhibitory concentration (IC50) of a GCV-sensitive strain by 13 fold (Chou and Marousek, 2006).

MBV competitively inhibits pUL97 (Biron et al., 2002) and blocks the phosphorylation of several downstream proteins including cellular components and the viral proteins pp65 and pUL44 the DNA polymerase accessory protein (Prichard, 2009). Like Cdc2/Cyclin-dependent kinase 1 (CDK1) in CMV-uninfected cells, the viral kinase pUL97 phosphorylates nuclear lamina components (lamin A/C), facilitating the removal of mature virions from the nucleus. Consequently, MBV treatment results in the accumulation of immature virions in the nucleus (Hamirally et al., 2009). It also inhibits CMV DNA replication through pUL44 inhibition. *In vitro* drug combination assays showed additive effect with foscarnet, and synergy with artesunate (Morère et al., 2015). Additive effect was also observed with cidofovir or letermovir (quinazoline), while association with BDCRB, a benzimidazole inhibitor of the terminase, or with sirolimus, a mTor inhibitor, was synergistic (Chou et al., 2019).

#### 2.2.2 Preclinical studies

*In vitro*, MBV has selective activity against CMV. Its activity has been demonstrated against Epstein–Barr virus (EBV), however it is not active against herpes simplex virus, varicella-zoster virus (VZV) or human herpesviruses 6 and 8 (HHV-6 and HHV-8) (Prichard, 2009).

Preclinical studies showed that MBV has a better oral bioavailability, a better safety profile and a lower toxicity for host cells than current drugs (GCV, FOS and CDV) with theoretical benefits for the viral inhibition and cross-resistances appearing (Lu and Thomas, 2000; Koszalka et al., 2002; Chou et al., 2012a). MBV also reached concentrations above EC_50_ at the fetal face when perfused across a third trimester placenta (Faure Bardon et al., 2020). In addition, it inhibits CMV replication in first trimester placental villi models in histoculture, with the same EC_50_ as *in vitro* (Morère et al., 2015).

In animal models, the oral bioavailability of MBV is 90% in rats and 50% in monkeys. MBV is excreted via the biliary route and, to a lesser extent, via the metabolic and renal pathways. The minimum effect dose in rats was 100 mg/kg/day and the no-effect dose in monkeys was 180 mg/kg/day (Wang et al., 2003). It was initially distributed in the gastrointestinal tract of rats, but did not cross the blood–brain barrier. This study showed favorable results for MBV’s safety profile (Koszalka et al., 2002). In addition, Kern et al. (2004a) demonstrated that oral MBV significantly reduced HCMV replication at concentrations of 75 mg/kg twice daily in SCID-humanized mice with human fetal retinal tissue implants or thymus/liver implants. However, MBV was more effective in treating thymus/liver infection, as it was shown to be poorly absorbed by ocular tissues.

#### 2.2.3 Clinical studies

Several Phase I clinical trials have been conducted with MBV to evaluate its safety, pharmacokinetics and efficacy against CMV infection. In fact, two Phase I clinical trials with escalating single doses of MBV (50 mg to 1,600 mg) were conducted in healthy and human immunodeficiency virus (HIV)-infected patients. 30–40% of an oral dose of MBV was absorbed, and C_max_ was reached 1–3 h after administration (Wang et al., 2003). MBV was rapidly eliminated. At the 400 mg dose, no statistical difference was observed whatever the renal functions of patients (Wang et al., 2003; Swan et al., 2007). The main side effect of MBV is dysgeusia. A Phase I study with multiple oral doses of MBV was carried out to evaluate its antiviral activity. MBV was administered orally at doses of 100 mg twice daily, 400 mg once daily or 400 mg twice daily to CMV-seropositive HSCT recipients. One hundred days after transplantation, pp65 antigenemia was lower in all groups than in the placebo group (15, 19, 15% vs. 39% respectively). In addition, pharmacokinetic analysis of the 400 mg twice-daily dose showed higher C_max_ and area under the curve (AUC) values than the 100 mg twice-daily dose, but with no improvement in antiviral activity and more side effects (Winston et al., 2008).

At low doses, MBV failed to meet the primary endpoints of the initial Phase III study for prophylaxis in hematopoietic stem cell allograft and liver transplant recipients. However, in a Phase II dose-ranging clinical trial, MBV ≥ 400 mg twice was active against refractory or resistant CMV infections in transplant recipients (Papanicolaou et al., 2019). This dosing also showed similar efficacy to those of valganciclovir in pre-emptive treatment of solid organ transplant and HSCT recipients (Maertens et al., 2020). MBV is mainly metabolized in the liver, and moderate hepatic impairment increased total MBV concentrations. This suggests that dose adjustment of MBV may not be necessary for individuals with mild to moderate hepatic impairment (Song et al., 2023).

A randomized Phase III trial, the Solstice study (NCT02931539), demonstrated the efficacy of MBV in SOT and HSCT patients with refractory CMV infections with or without resistance. The study was conducted on 352 patients (235 patients receiving MBV 400 mg twice daily versus 117 patients receiving investigator-assigned therapy (IAT): GCV, VGCV, FOS or CDV) for 8 weeks with a 12-week follow-up. Endpoints were CMV disappearance at the end of week 8 and MBV disappearance and symptom control at the end of week 8, maintained until week 16. Significantly, more patients in the MBV group achieved the primary endpoint (55.7% vs. 23.9%; *p* < 0.001) and the secondary endpoint (18.7% vs. 10.3%; *p* < 0.01). Side effects were less frequent in the MBV group than in the IAT group. Acute kidney injury was more frequent in patients treated with FOS (21.3% vs. 8.5%), and neutropenia was more frequent in patients treated with GCV/GCV (9.4% vs. 33.9%). In the MBV group, 13.2% of patients discontinued treatment due to drug-related adverse events, compared with 31.9% in the IAT group (Avery et al., 2021).

#### 2.2.4 Resistance

**Figure 4:**
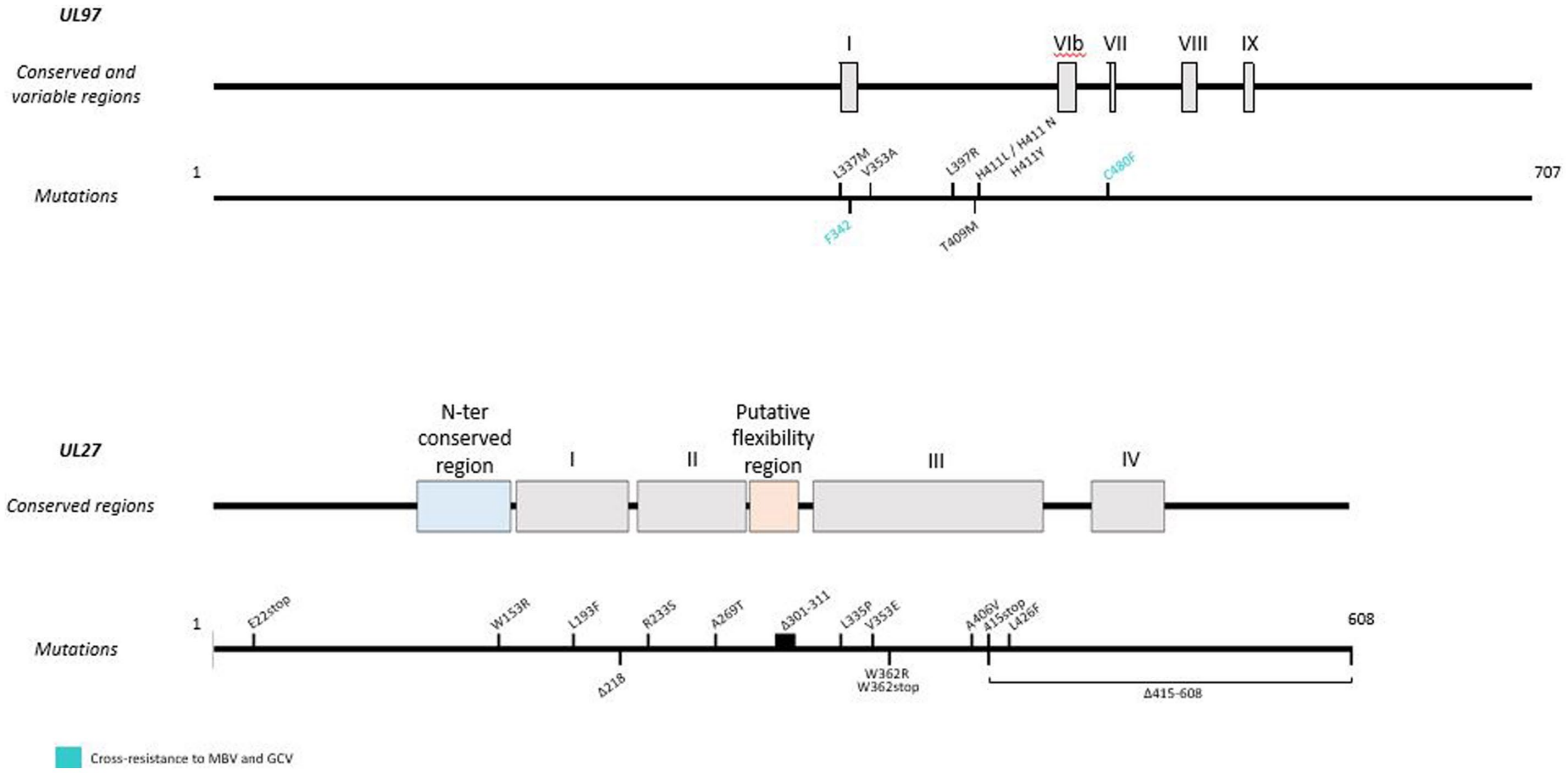
Resistance mutations to maribavir in *UL97* and *UL27* genes. Mutations responsible for cross-resistance with GCV are represented in blue. Mutations are referenced on genes under the map of conserved and variable regions. Scaled representation.

Although MBV is a newly approved antiviral, resistance mutations (Figure 4) have already been found in viral genes *UL97* and *UL27*. Indeed, some pUL97 mutations (V353A, L397R, L337M, T409M, H411L, H411N, H411Y, F342, C480F) confer moderate to high-level resistance to MBV, with a 3.5 to 200 fold increase of the EC_50_ (Chou et al., 2007, 2019; Chou and Marousek, 2008; Chou, 2020). These mutations are close to the kinase ATP-binding and catalytic domains upstream the GCV resistance mutations (Chou and Marousek, 2008). Mutations F342Y and C480F are responsible for cross-resistance to ganciclovir and may be present before MBV treatment (Chou et al., 2023). In addition, mutations in the UL27 gene confer low resistance to MBV with a 2- to 3-fold increase in EC50. These mutations (R233S, W362R, W153R, L193F, A269T, V353E, L426F, E22stop, W362stop, 218delC, and 301-311del) compensate for pUL97 inhibition by destabilizing Tip60 (histone acetyltransferase), increase p21 expression and inhibit cyclin-dependent cellular kinases (Chou et al., 2004; Chou, 2009; Kamil and Coen, 2011; Reitsma et al., 2011). In phase II and phase III clinical trials, resistance to MBV emerged in 52 and 26% of treated patients, respectively (Papanicolaou et al., 2019; Chou et al., 2023).

## 3 New molecules with activity against CMV

### 3.1 Direct-acting antivirals

#### 3.1.1 Brincidofovir

Brincidofovir (BCV, CMX001; HDP-CDV) [[(S)-2-(4-amino-2-oxo-1(2H)-pyrimidinyl)-1-(hydroxymethyl)ethoxy]methyl]mono[3-(hexadecyloxy)propyl] ester (Figure 2) developed by Chimerix is a lipid antiviral conjugate (LAC) composed of a lipid [1-0-hexadecyl-oxypropyl (HDP)] covalently linked to the acyclic nucleotide analog CDV, enabling the drug to utilize the natural absorption pathways of lysophosphatidylcholine in the small intestine (i.e., passive diffusion and flipases; Lanier et al., 2016). Currently, the United-States FDA approves BCV for treatment of smallpox.

#### 3.1.2 Mechanism of action

**Figure 5:**
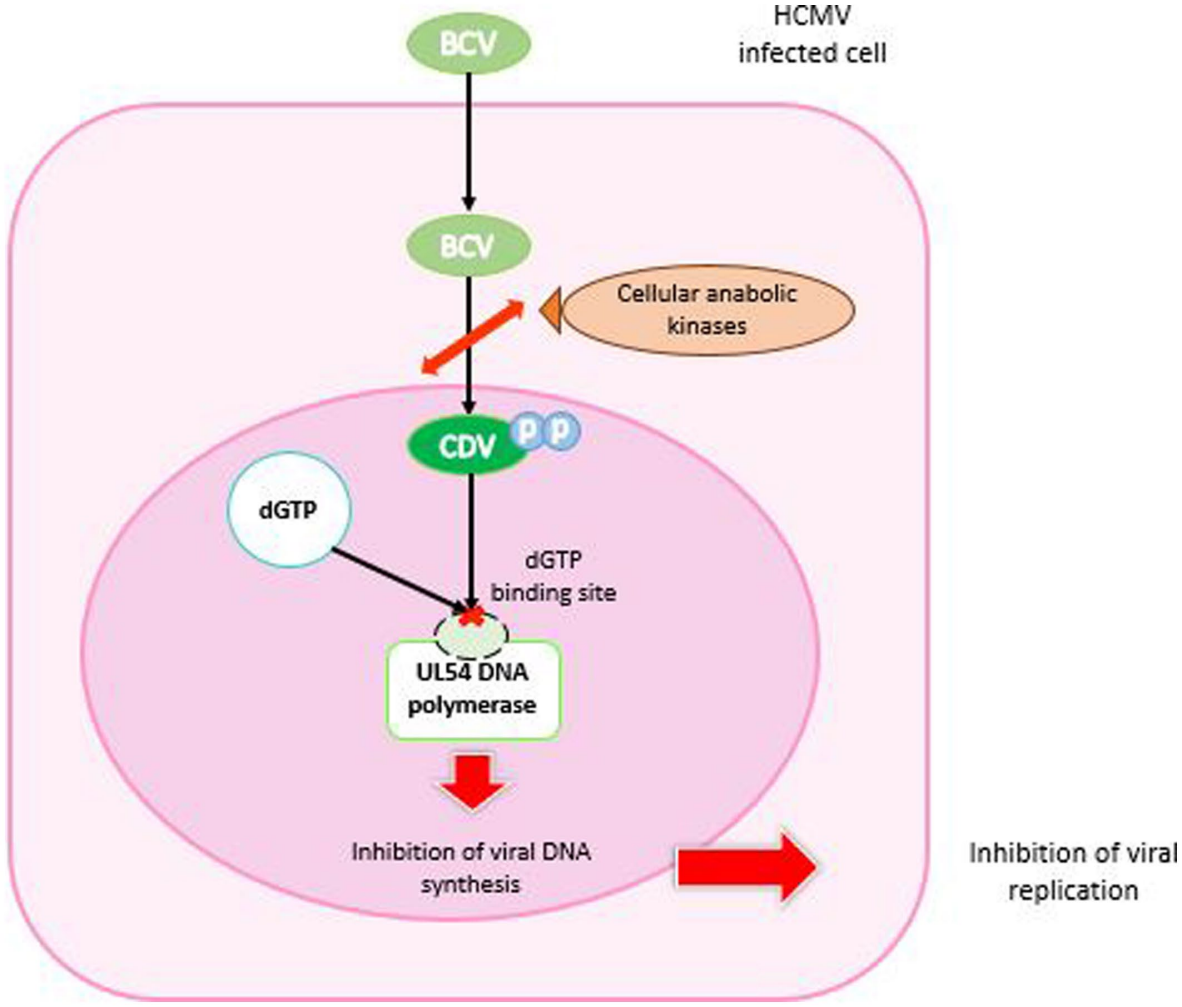
Mechanism of action of brincidofovir. BCV enters the HCMV-infected cell, is cleaved and phosphorylated by cellular anabolic kinases to cidofovir diphosphate. In the nucleus, CDV-PP binds competitively to the dGTP binding site of UL54 DNA polymerase. The result is inhibition of DNA synthesis and arrest of viral replication.

BCV was designed to remain intact in plasma and deliver the drug directly to target cells. It enabled enhanced cellular uptake and high intracellular levels of the converted active antiviral agent, CDV-diphosphate (CDV-PP), increasing antiviral activity against CMV by 2 to 3 orders of magnitude compared with CDV alone (Aldern et al., 2003; Williams-Aziz et al., 2005). BCV is cleaved to release CDV. Then, CDV is converted by intracellular anabolic kinases to CDV-PP, the active inhibitor of viral DNA synthesis. Unlike CDV, BCV is not a substrate for the human organic anion transporter 1, which mechanistically explains the absence of renal toxicity observed in clinical trials with BCV (Tippin et al., 2016; Figure 5).

#### 3.1.3 Preclinical tests

BCV has been developed for the treatment of infections by double-stranded DNA viruses. It has broad-spectrum efficacy against herpesviruses, polyomaviruses, adenoviruses, papillomaviruses and orthopoxviruses (Beadle et al., 2002; Bidanset et al., 2004; Williams-Aziz et al., 2005). BCV is effective against clinical isolates of HCMV (EC50 of 0.0009 μM against HCMV strain AD169) and HSV, including isolates resistant to GCV and ACV (Williams-Aziz et al., 2005; James et al., 2013). In addition, BCV has been shown to be 10 to 100 times more active than CDV against murine CMV (Kern et al., 2004b).

*In vivo* tests were carried out on animal models to evaluate its efficacy in congenital CMV infection. BCV showed antiviral activity of 0.004 μM ± 0.001 μM against guinea pig CMV (GPCMV). At the end of the second or beginning of the third trimester of gestation, guinea pigs were infected with GPCMV. Significant pup survival was observed in the BCV group (93–100% vs. 50–60%; *p* ≤ 0.019). Viral load was significantly reduced in the spleen and liver of pups after BCV treatment (*p* = 0.017 and *p* = 0.029 respectively). Although pup survival was improved with 4 mg/kg treatment, virus levels in fetal tissues were related to those in control tissues. This suggests that BCV could have been a good candidate for the treatment of congenital CMV infections in humans, with high tolerance (Bravo et al., 2011).

#### 3.1.4 Clinical trials

Painter et al. evaluated the pharmacokinetics and safety of BCV in the first Phase I clinical trial in 2012. This was a randomized, double-blind, placebo-controlled, parallel-group, dose-escalation trial in healthy adults. There were no adverse events during the trial. No significant changes in pharmacokinetic parameters were reported. Gastrointestinal analyzes showed no BCV-related mucosal changes. After multiple doses, no accumulation of BCV was observed. Maximum plasma concentrations of BCV were observed 2 to 3 h after dosing. This trial showed that BCV was relatively well tolerated and had a high bioavailability with a dose of approximately 140 mg in adults (2 mg/kg) (Painter et al., 2012).

From 2009 to 2011, Marty et al. (2013) conducted a phase II clinical trial (NCT00942305) on 230 adult CMV-seropositive hematopoietic stem cell transplant recipients at 27 centers. Five sequential study cohorts were planned according to a double-blind ascending dose schedule (3:1 ratio to receive BCV or matching placebo). Drugs were administered for 9 to 11 weeks post-transplant. The primary endpoint was a CMV-related event, i.e., CMV disease or a plasma CMV DNA level above 200 copies/ml. In the BCV 100 mg twice weekly group, the incidence of CMV-related events was significantly lower than in the placebo group (10% vs. 37%; *p* = 0.002). The most frequent side effect of treatment was diarrhea in the BCV 200 mg weekly group. There were no reports of myelosuppression or nephrotoxicity (Marty et al., 2013).

The Phase III clinical trial (NCT01769170) was a randomized, double-blind, placebo-controlled (2:1) trial for CMV prophylaxis in 452 CMV-seropositive adults with HCT without CMV viremia. Patients received BCV or placebo until week 14 after HCT. The primary endpoint was the proportion of patients who developed clinically significant CMV infection (CS-CMVi: CMV viremia requiring preemptive treatment or CMV disease) up to week 24 after HCT. This proportion was similar in both groups (51.2% vs. 52.3%; *p* = 0.805). Fewer BCV-treated patients developed CMV viremia up to week 14 than placebo-treated patients (41.6%; *p* < 0.001). BCV resulted in more frequent adverse events (51.1% vs. 37.6%) such as acute graft-versus-host disease (32.3% vs. 6.0%) and diarrhea (6.9% vs. 2.7%). All-cause mortality at week 24 was 15.5 and 10.1% in the BCV and placebo groups, respectively. In conclusion, BCV did not reduce CMV viremia at week 24 post-transplant, and was associated with gastrointestinal toxicities (Marty et al., 2019). BCV is not available to date for the treatment of CMV infection.

#### 3.1.5 Resistance

Since 2013, CDV resistance mutations in UL54 DNA polymerase have been shown to confer resistance to BCV. *In vitro*, increasing concentrations of BCV over 10 months conferred CDV and BCV resistance on a wild-type strain. Genotyping of the strain revealed a D542E mutation in pUL54, which was responsible for a more than 10-fold reduction in susceptibility to BCV and CDV in marker-transfer experiments. This mutation did not confer resistance to GCV or FOS. A smaller plaque phenotype and slower replication kinetics than the parent viruses were also demonstrated. This is the first mutation described under BCV selective pressure. This suggests that BCV may have a unique resistance profile associated with reduced viral replication and maintenance of sensitivity to FOS and GCV (James et al., 2013).

*In vitro* experiments under BCV pressure selected the N408K and V812L mutations in CMV DNA polymerase, which were already known to confer resistance to CDV. In addition, new substitutions in the exonuclease domain were identified: D413Y, E303D and E303G, which confer resistance to GCV and CDV, with 6- to 11-fold resistance to BCV, or 17-fold when E303G is combined with V812L. This confirmed the expected pattern of cross-resistance (Chou et al., 2016).

In a clinical study, Lanier et al. investigated CMV genotypes from a Phase II trial comparing BCV to placebo for prophylaxis of CMV infections in HCT recipients. Two mutations (M827I and R1052C) were reported in pUL54 in a small number of patients, but did not confer resistance to BCV, CDV, GCV or FOS. This study suggests that the first-line use of BCV for the prevention of CMV infection may preserve downstream options for patients (Lanier et al., 2016). Nevertheless, A987G and F412L mutations in pUL54 have been reported in other studies using BCV as rescue therapy (Kaul et al., 2011; Vial et al., 2017). These mutations were known to confer resistance to CDV, suggesting that the emergence of resistance could occur after BCV treatment.

### 3.2 Metylenecyclopropane analog: cyclopropavir

Cyclopropavir (CPV, Filociclovir (FCV), ZSM-I-62), (Z)-9-{[2,2-bis-(hydroxymethyl)cyclopropylidene]methyl}guanine (Figure 2) is a new analog of methylenecyclopropane (MCPN) (Zhou et al., 2004). This antiviral showed a good activity against HCMV and murine CMV in animal models (Kern et al., 2004b). In addition, CPV proved highly potent against HCMV (wild-type and GCV-resistant mutants in pUL97 and pUL54), EBV, both variants of HHV-6, HHV-7 and HHV-8 (Kern et al., 2005).

#### 3.2.1 Mechanism of action

**Figure 6:**
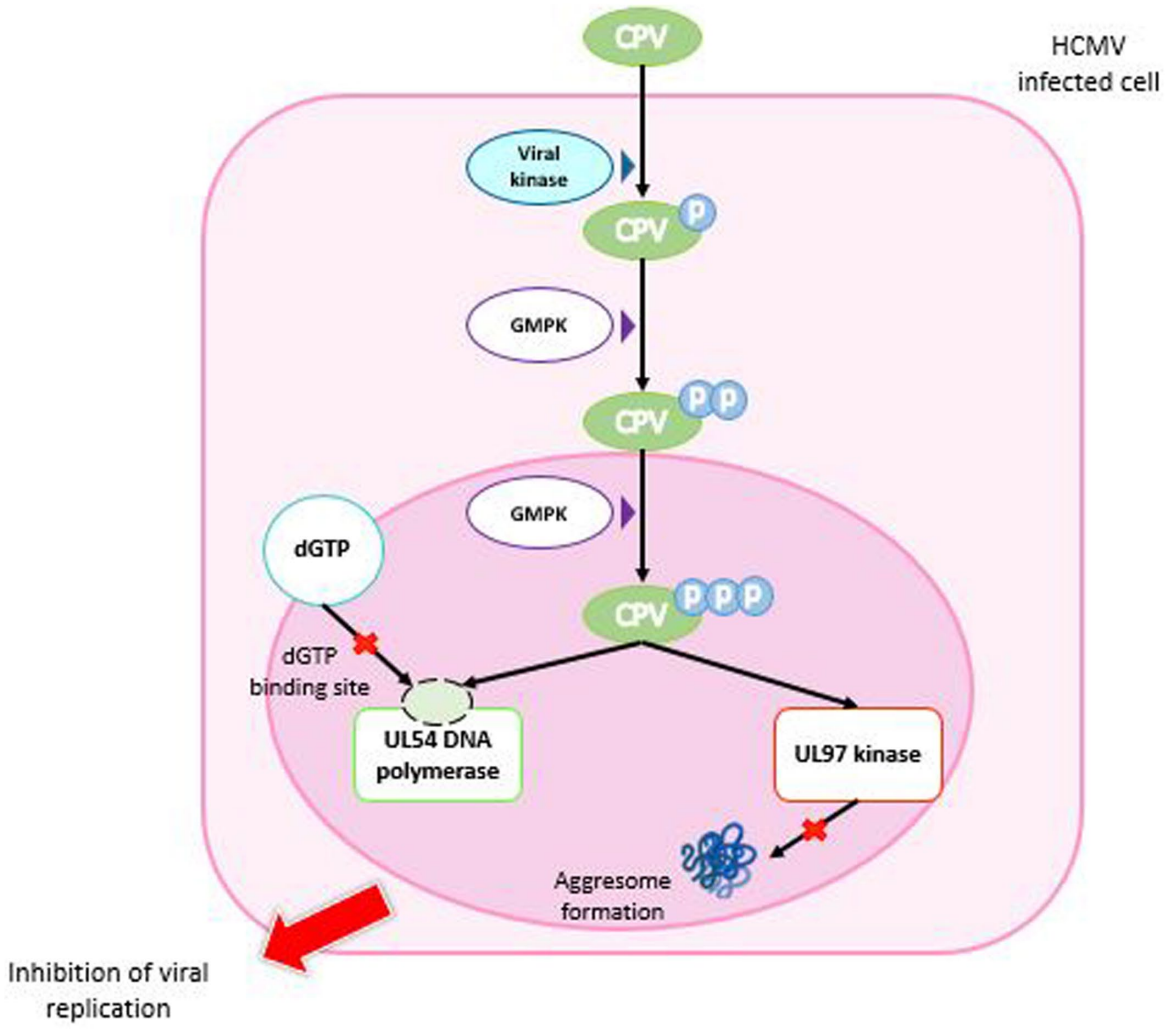
Mechanism of action of cyclopropavir. CPV enters the HCMV-infected cell and is phosphorylated by a viral kinase to CPV monophosphate. Two successive phosphorylations by GMPK are required to obtain CPV triphosphate. In the nucleus, CPV-triP binds competitively to the dGTP binding site of DNA polymerase UL54 and inhibits DNA synthesis. It also inhibits the ability of the viral UL97 kinase to prevent aggresome formation. Both mechanisms lead to inhibition of viral replication. GMPK: guanosine monophosphate kinase.

As with other nucleoside analogs such as GCV, activation of CPV by tri-phosphorylation is required (Littler et al., 1992). The primary phosphorylation is performed by the viral kinase pUL97 (HCMV) or pU69 (HHV-6), whereas the second and third phosphorylations are made by the guanosine monophosphate kinase (GMPK), thus resulting in tri-phosphate CPV (CPV-TP) (Kern et al., 2005; Gentry et al., 2011; Komazin-Meredith et al., 2014). Therefore, the conversion mechanism of CPV is different from that of GCV; it necessitates a single cellular enzyme to have CPV-triphosphate (CPV-TP; Figure 6).

CPV inhibits HCMV replication by a dual mechanism, inhibiting both pUL54 DNA polymerase and UL97 kinase (James et al., 2011). Indeed, some mutations on pUL54 confer a resistance to CPV, which confirms that the viral polymerase is a target of CPV (Chou et al., 2012b). CPV-TP inhibits pUL54 by competition with dGTP and takes place as chain terminator that stops the DNA synthesis. Interestingly, of the two CPV enantiomers, (+)-CPV-TP could have a twenty-fold higher affinity with pUL54 (Chen et al., 2016). In addition, (+)-CPV is preferencially converted in (+)-CPV-TP than (−)-CPV-TP by the GMP kinase (Gentry et al., 2011). Besides this mechanism, the inhibition of normal function of pUL97 kinase by CPV was assessed by cell transfection with plasmids expressing pUL97 and a reporter plasmid expressing pp65-GFP. CPV prevented the pUL97 capacity to inhibit aggresomes formation, as MBV (James et al., 2011).

#### 3.2.2 Preclinical tests

A study on immunocompromised SCID mice (BALB/c) infected with MCMV, orally administered CPV showed a good effectiveness compared with GCV. Mortality rates were significantly reduced with CPV. Indeed, reducing of viral replication was much more effective in CMV target organs like liver, spleen and lung (Kern et al., 2004c).

Additionally, CPV showed a greater efficacy *in vitro* and *in vivo* than GCV without any increase of toxicity (Zhou et al., 2004; Kern et al., 2005) and achieved therapeutic concentrations *in vivo* without prodrug modification (Wu et al., 2009). In addition, CPV was used in combination with BDCRB and produced a statistically significant synergistic effect against HCMV *in vitro* (O’Brien et al., 2018). On the other hand, the introduction of 1.0 or 10 nM MBV demonstrated a competitive inhibition of CPV phosphorylation with a Ki of 3.0 ± 0.3 nM (Gentry et al., 2010).

Pharmacokinetics, toxicokinetics and absorption, distribution, metabolism and excretion (ADME) datas of CPV showed good results in animals. CPV has a high oral biodisponibility. It also shown that plasma concentration were higher after the first dose of CPV than after the fourteenth daily dose. CPV did not induce or inhibit cytochrome P450 and was minimily metabolized by liver microsomes (Brooks and Bowlin, 2013).

The safety study showed that CPV did not present adverse effects on central nervous system, respiratory and cardiovasular systems. Besides, toxicology studies demonstrated that CPV did not cause haemolysis *ex vivo* (Brooks and Bowlin, 2013).

#### 3.2.3 Clinical trials

The first phase 1A clinical trial (NTC01433835) included 48 healthy adults (3 males, 45 females) with a main age of 50.3 years. This randomized, placebo-controlled (3:1) trial evaluated CPV safety and pharmacokinetics after the administration of various doses ranging from 35 to 1,350 mg. No serious adverse effects were classified. C_max_ were reached after 1 to 2 h after oral administration and the CPV was not detectable 24 h after last dose of treatment (Brooks et al., 2015).

A phase 1B, double blind, randomized, placebo-controlled (3:1), single center, multiple ascending doses, clinical trial (NCT02454699) was done to assess safety, tolerability and pharmacokinetics of CPV at various doses in 24 healthy adult volunteers monitored for 22 days (7 males and 17 females; main age 47.4 years). Doses of CPV were 100, 350 and 750 mg for 7 days. During this study, no serious adverse effect was highlighted. Indeed, main adverse events concerned gastrointestinal tract (17%), nervous system (11%) and skin and subcutaneous tissues (11%). The only severe adverse event appeared in the 750-mg cohort was a reversible grade 3 elevation in serum creatinine and bilirubin associated with a 1-log increase of CPV in plasma after 24 h of the initial dose. The C_max_ was reached at 2 to 3 h following administration and CPV was undetectable in plasma 24 h after the last dose, as phase 1A trial. Finally, authors concluded that, *in vivo*, doses as low as 100 mg were sufficient to inhibit CMV (Rouphael et al., 2019).

So far, no phase II or III clinical trials are in progress (source: ClinicaTrials.gov).

#### 3.2.4 A new antiviral against adenoviruses

Adenoviruses are responsible for a variety of infections in children, and can cause acute hepatitis with high morbidity and mortality (Kajon and St George, 2022). Recent studies have also demonstrated the antiviral activity of CPV against adenovirus (HAdV) by inhibition of the adenovirus-encoded DNA polymerase (Toth et al., 2020). Hartline et al. (2018), have shown that the strain human adenovirus type 5 (HAdV5) of the American Type Culture Collection (ATCC) was sensitive to CPV. CPV has shown a high potential to inhibit *in vitro* HAdV replication with a higher efficacy than CDV (5- to 10- fold higher) (Tollefson et al., 2022).

The same potential against adenoviruses has been observed with BCV. It has been demonstrated in several clinical trials, notably in the treatment of severe adenovirus infection and disease in 2022 (NTC02596997) (Alvarez-Cardona et al., 2020; Chimerix, 2022).

#### 3.2.5 Resistance

**Figure 7:**
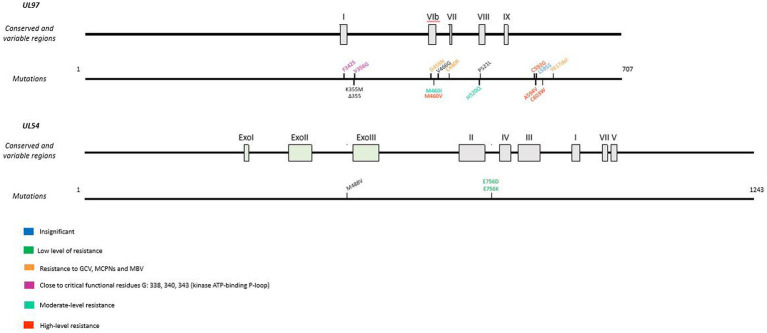
Resistance mutations to cyclopropavir in UL97 and UL54 genes. CPV resistance mutations according to EC50 values. In *UL97*, cross-resistance mutations are represented in orange and mutations next to the ATP-binding P-loop are in pink. Mutations are referenced on genes under the map of conserved and variable regions. Scaled representation.

Mutations are known to confer CPV resistance (Figure 7). Indeed, a recombinant virus with Δ498 bp mutation in the *UL97* open reading frame (ORF) is resulting in a protein with a missing kinase domain (Gentry et al., 2013). The K355M mutation confers to the virus a moderate 5- to 7- fold increase in EC_50_ versus a 13- to 25- fold increase for FCV and GCV, respectively, (Komazin-Meredith et al., 2014). Recombinant virus lacking pUL97 kinase domain is 20 times more resistant to CPV (Kern et al., 2005), which can be explained by the need for the first phosphorylation of CPV by pUL97, essential for activation of the molecule (Gentry et al., 2013). Mutations have been identified *in vitro*, ranging from insignificant mutations to resistance mutation confering cross-resistance to GCV, MBV and CPV.

Mutations close to critical functional residues were also identified: F342S and V356G. These mutations are close to the highly conserved residues involved in the kinase ATP-binding P-loop G338, G340 and G343 (Chou et al., 2013). The L595S mutants are resistant to GCV but remain susceptible to CPV (Chou and Bowlin, 2011). On the other hand, M460V, A594V, C592G and C603W, which confer low resistance to GCV, increase EC50 by 3 to 5-fold; M460I and H520Q induce high resistance to CPV, with a 12- to 20-fold increase in EC50 (Chou and Bowlin, 2011; James et al., 2011).

In addition, D456N, C480R and Δ617 confer resistance to GCV, MBV and all the MCPNs including CPV (Komazin-Meredith et al., 2014).

The combination H520Q-M488V (UL97-UL54) induced the highest level of resistance to CPV. Resistance mutations are close to finger and palm domains of the polymerase catalytic core (Chou et al., 2012b). Two other mutations in pUL54 were shown to confer a lower resistance to CPV: E756D and E756K. Both are involved in FOS resistance (Lurain and Chou, 2010).

### 3.3 Benzimidazole analogs: BDCRB, TCRB and tomeglovir

BDCRB or 2-Bromo-5,6-dichloro-1-(beta-d-ribofuranosyl)benzimidazole and TRCB or 2,5,6-Trichloro-1-(beta-D-ribofuranosyl)benzimidazole (Figure 2) are benzimidazole ribofuranoside. These two molecules are potent and selective inhibitors of HCMV replication (Townsend et al., 1995). Tomeglovir (BAY 38–4,766) or {3-hydroxy-2,2-dimethyl-N[4({[5-(dimethylamino)-1-naphthyl]sulfonyl}amino)-phenyl] propanamide} (Figure 2) is an oral, non-nucleoside compound related to the D-benzimidazole ribonucleosides. It is a potent and selective inhibitor of HCMV replication by inhibition of viral DNA concatemers processing (Reefschlaeger et al., 2001; Weber et al., 2001).

#### 3.3.1 Mechanism of action

**Figure 8:**
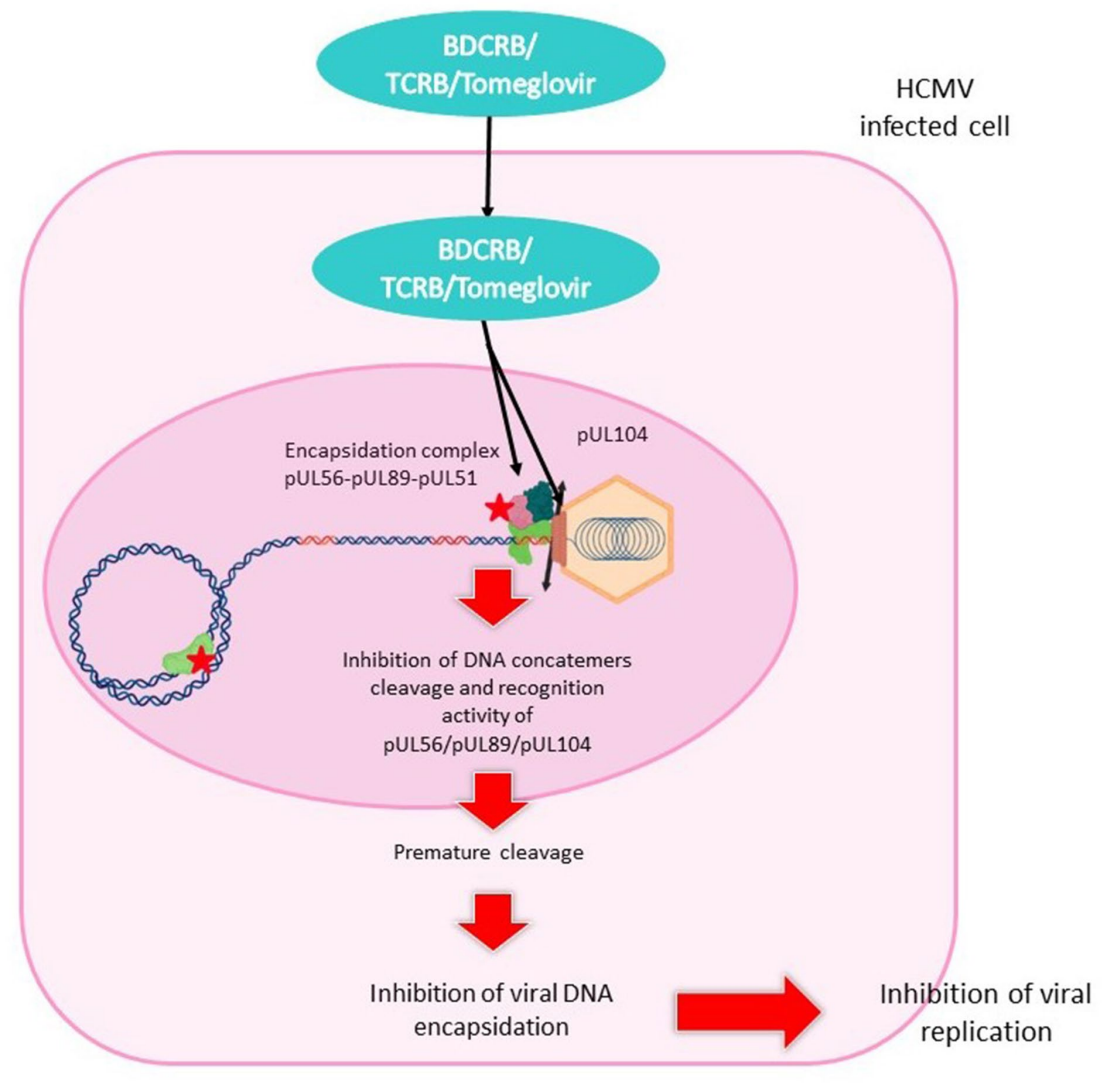
Mechanism of action of benzimidazol analogs. BDCRB, TCRB and tomeglovir enter the HCMV-infected cell and travel to the nucleus. They inhibit the encapsidation complex composed of pUL56, pUL89 and pUL51, and inhibit the portal protein pUL104. This mechanism leads to inhibition of DNA cleavage and concatemer recognition, or to premature cleavage. Encapsidation is interrupted and viral replication halted.

The mechanism of action of BDCRB and TCRB (Figure 8) does not need phosphorylation at the 5′ position and does not involve the inhibition of DNA synthesis (Krosky et al., 2002). It prevents the cleavage of high molecular weight viral DNA concatemers to monomeric genomic lengths (Underwood et al., 1998). Resistance mutations were found in HCMV genes *UL56* and *UL89* suggesting that BDCRB and TCRB target the encapsidation step (Krosky et al., 1998; Underwood et al., 1998). BDCRB partially inhibits the ATPase activity of pUL56 and the pUL89-associated nuclease activity at high concentrations (Scholz et al., 2003). Furthermore, it has been suggested that BDCRB causes HCMV terminase to skip the normal cleavage site and continue packing DNA until a second cleavage site is encountered 30 kb further (McVoy and Nixon, 2005). In GPCMV, monomer-length genomes are plentifully produced under BDCRB, but are slightly truncated at the left end (Nixon and McVoy, 2004). This is in accordance with the fact that BDCRB alters recognition and cleavage of DNA by the terminase complex.

Tomeglovir mode of action (Figure 7) is the same as that of BDCRB and TCRB described above. This molecule targets HCMV-specific proteins required for cleavage and packaging of viral DNA, transforming high molecular weight viral DNA concatemers into monomeric genomes length (Reefschlaeger et al., 2001).

#### 3.3.2 Preclinical tests

BDCRB and TCRB were effective against HCMV with EC_50_ of 0.7 μM and 2.9 μM in plaque assays, respectively. It was also proved that BDCRB and TCRB were inactive against HSV-1. However, the incorporation of Cl and Br into these two molecules improved dramatically their therapeutic index (Townsend et al., 1995). It was also shown that BDCRB is inactive against HHV-6 and HHV-7 (Yoshida et al., 1998). Another study demonstrated the inefficacy of BDCRB against HSV-1, HSV-2, VZV, HHV-8 but an activity against EBV (Williams et al., 2003). Furthermore, it was demonstrated that conformational changes in BDCRB structure could increase its spectrum against herpesviruses. Indeed, the L-analog of BDCRB was effective against HHV-6 (Prichard et al., 2011). Tomeglovir was effective against HCMV strains (EC_50_ = 0.52 ± 0.014 μM) (Reefschlaeger et al., 2001). GCV-resistant clinical isolates were also susceptible to tomeglovir (McSharry et al., 2001).

Interestingly, HCMV strain AD169 was more sensitive to benzimidazole than the strain Towne (Krosky et al., 2000). Although TCRB and BDCRB showed excellent activity *in vitro*, their glycosidic bonds are hydrolyzed *in vivo* to less active metabolites that reduce their activity (Good et al., 1994).

In addition, BDCRB showed synergy in combination with MBV, synergy at low concentrations and antagonism at higher concentrations with tomeglovir (Evers et al., 2002). Furthermore, tomeglovir had an antagonistic effect when combined with GCV (Evers et al., 2002).

The toxicity of benzimidazole analogs was tested in bone marrow cells. One hundred μM BDCRB inhibited cell proliferation by 20% over a 10-day period, while 100 μM GCV inhibited it by 52%. In other experiments on hematopoietic progenitor cell colony-forming assays, 100 μM BDCRB affected BFU-E and CFU-GM (burst forming units-erythroid and colony forming units-granulocyte/macrophage) by 31 and 47%, respectively. In contrast, GCV inhibited BFU-E by 54% and CFU-GM by 86%. However, TCRB was less effective than BDCRB. This study concludes that certain benzimidazole nucleosides are less toxic than conventional drugs (Reza Nassiri et al., 1996).

Additionally, *in vitro* study with BDCRB in guinea pig embryo or lung fibroblasts showed that GPCMV was sensitive to BDCRB (EC_50_ = 4.7 μM). BDCRB did not inhibit the formation of genome-sized GPCMV DNA, which was packaged but not protected from nuclease. Termini formed on GPCMV genome were altered by BDCRB. Then, BDCRB participated to the retention of C capsids in the nucleus (McVoy and Nixon, 2005). However, more recently, a GPCMV resistant to BDRCRB was generated and characterized. Genetic alterations were reported: an L406P substitution in GP89, the HCMV UL89 homolog; a 13.4 kb internal deletion of the GP131-GP143 non-essential ORFs; and a dramatic increase in the number of iterations of a 1 kb terminal repetitive sequence, from 0 or 1 to up to 9 at either genomic end (Ourahmane et al., 2018).

In animal model experiments, efficacy was evaluated in an ocular model of SCID-humanized mice infected with the Toledo strain of HCMV. BDCRB was administered at doses of 50 mg/kg for 28 days or 25 mg/kg twice daily for 1 week and once daily for 2 weeks. A slight but non-significant reduction in HCMV titers was observed in the 50 mg/kg group, and no reduction in mean titers was observed in the 25 mg/kg group. These results showed that BDCRB could only be active against HCMV at high concentrations. In the second experiment, HCMV-infected SCID-humanized retinal implants were treated with BDCRB or CDV for 28 days. Mice were treated with 75 mg/kg BDCRB from day one post-infection. BDCRB had no effect on reducing viral titers in retinal implant tissues. These results demonstrated the ineffectiveness of BDCRB in crossing the blood-ocular barrier in *in vivo* models. In addition, the same experiments were carried out with visceral organs (fetal thymus and liver) implanted in the kidney capsule. Doses of BDCRB blocked viral replication by around 2 to 3 log^10^ PFU/g (Kern et al., 2004a).

Tomeglovir was evaluated in MCMV-infected immunodeficient mice and reduced viral load in target organs in a manner comparable to GCV. Weight loss (consequence of viral infection) is reduced after tomeglovir administration (Reefschlaeger et al., 2001). Another study on murine model with *per os* administration of tomeglovir at dose ≥10 mg/kg showed similar results (Weber et al., 2001). A study in guinea pigs also demonstrated that peak plasma tomeglovir levels were 26.7 mg/mL 1 h after dosing. It reduced both viremia and DNAemia, as well as mortality following lethal GPCMV challenge in immunocompromised guinea pigs, from 83 to 17% (*p* < 0.0001). This study demonstrated the safety, pharmacokinetics and favorable therapeutic profiles of tomeglovir (Schleiss et al., 2005).

#### 3.3.3 Clinical trials

Currently, no published clinical trial was performed to assess BDCRB, TCRB and tomeglovir in human (source: ClinicaTrials.gov).

#### 3.3.4 Resistance

Resistance mutations to BDRCRB and TCRB (Table 2) are located in pUL56 (Q204R) and in pUL89 (D344E and A355T). If combined, these mutations showed a greater resistance to benzimidazole analogs than alone. Nevertheless, these mutations did not confer resistance to GCV (Krosky et al., 1998; Underwood et al., 1998; Evers et al., 2002). Additionally, mutations M360I in M89 exon II and P202A and I208N in M56 confer murine CMV resistance to tomeglovir. Mutation in M89 exon II had analogous mutations in HCMV pUL89 mentioned for BDCRB and TCRB but those in M56 did not have it in HCMV pUL56. Thus, pUL89 could be directly targeted by tomeglovir and pUL56 could compensate for restricted activities of Buerger et al. (2001). More recently, Chou (2017) highlighted new mutations in *UL89* gene and *UL56* responsible for tomeglovir resistance. In pUL89 N329S, T350M, H389N, N405D, D344E, C347S and V362M conferred moderate to high drug resistance. The mutation I334V did not conferred tomeglovir resistance but affected growth fitness when combined with N405D. Then, in pUL56, Q204R was shown as lower-grade resistance mutation (Chou, 2017).

**Table 2:** Resistance mutations to benzimidazol analogs in UL56 and UL89 genes.

Gene		Resistance EC_50_ (μm; Fold change)			Reference
UL56	UL89	BDCRB	Tomeglovir	TCRB	
Q204R		**17 (14)**	**1.2 (2.7)**	**57 (23)**	Chou (2017), Krosky et al. (1998), and Evers et al. (2002)
L208M			**0.8 (3.4)**		Chou (2017)
N232Y			**1.2 (2.7)**		Chou (2017)
E407D			**1.3 (6.0)**		Chou (2017)
H637Q			**0.9 (2.0)**		Chou (2017)
V639M			**4.6 (10)**		Chou (2017)
L764M			0.19 (0.4)		Chou (2017)
	Q11H				
	I334V		1.1 (0.9)		Chou (2017)
	N320H		**3.0 (6.5)**		
	N329S		**2.0 (15)**		Chou (2017)
	D344E	**20,3 (10)**	**1.8 (1.7)**	**6–18 (10)**	Chou (2017), Underwood et al. (1998), and Krosky et al. (1998)
	C347S		0.6 (0.3)		Chou (2017)
	T350M		**2.8 (8.7)**		Chou (2017)
	A355T				
	M359I		**3.4 (7.4)**		Chou (2017)
	V362M		**1.2 (98)**		Chou (2017)
	H389N		**1.1 (29)**		Chou (2017)
	N405D		**6.9 (15)**		Chou (2017)
	I334V-N405D		**5.6 (12)**		Chou (2017)
	D344E-A355T	**20 (30)**		**>50**	Underwood et al. (1998)
H637Q-V639M			**7.5 (17)**		Chou (2017)
Q204R	D344E	**32 (13)**	**2.5 (5.8)**	**68 (30)**	Chou (2017) and Krosky et al. (1998)
F261L	D344E		**0.91 (2.1)**		Chou (2017)
M329T	D344E		**0.98 (2.2)**		Chou (2017)

Bold values are significant values for resistance.

The L406P mutation described in GPCMV QP89 was more than 50 residues away from the positions of the confirmed resistance mutations in HCMV pUL89. This mutation does not confer significant resistance of GPCMV to BDCRB but may have a compensatory function in enhancing replication by making easier genomic cleavage at cleavage sites containing multiple repeats. Furthermore, deletion of the E region of *HindIII* is unlikely to contribute directly to resistance to BDCRB. Thus, the accumulation of terminal repeats could be a response to BDCRB pressure and the resulting increase in genome length resulted in compensatory deletion of the *HindIII* E region (Ourahmane et al., 2018).

BDCRB and TCRB do not show any cross-resistance with GCV because of their different mechanisms of action and their different gene targets. Surprisingly, a cross-resistance with MBV is responsible for an increase of 2-3-fold EC_50_ even if these molecules do not have the same mechanism of action. Indeed, BDCRB and TCRB are DNA processing inhibitors and MBV is a DNA synthesis inhibitor (Evers et al., 2002).

In addition, a mutation was described in pUL104, the portal protein of HCMV, which is colocalized with pUL56, in resistant strains to benzimidazole nucleosides. However, this L21F pUL104 mutation alone did not prove sufficient to ensure resistance of HCMV to BDCRB (Komazin et al., 2004; Table 2).

## 4 Host-targeting antivirals

Several molecules targeting cellular metabolism have antiviral activity by interfering with cellular components participating in the viral replication cycle. These components have various targets, efficacy, and share the absence of identified viral resistance.

### 4.1 Artemisinin derivatives: artesunate, artemisone, and TF27

Artemisinin is an antimalarial drug that is an active compound of *A. annua* (1972). Many derivatives of this drug were developed. In this context, Saokim Ltd. (Hanoi, Vietnam) synthesized artesunate (Figure 2), a semisynthetic drug of artemisinin. This compound is available as intravenous or oral formulation to treat life-threatening malaria access. In 2010, the World Health Organization (WHO) recommended this drug as quinine to treat severe malarial infections during the first trimester of pregnancy (McGready et al., 2012; Roussel et al., 2017). Besides this activity, artesunate (ART) was shown as a good inhibitor of HCMV infections *in vitro* (Efferth et al., 2002, 2008). The laboratory of Sensitive Biology Therapy (S.B.T) synthesized its trimeric derived compound, TF27 (Figure 2; Hutterer et al., 2015; Reiter et al., 2015; Hahn et al., 2018). Recently, LH54, the heterologous hybrid compound of artesunate was developed in the laboratory of S.B.T. and has also a good antiviral activity (Wild et al., 2020). All these artesunate derivatives have shown good efficacy.

#### 4.1.1 Mechanism of action

Artemisin and its derivatives are primarily metabolized in dihydroartemisin (DHA) by cytochrome P-450 monooxygenase enzyme (CYP) 2B6 in human liver microsomes. This step is followed by the conversion into inactive metabolites via other enzyme systems. DHA has a half-life of about 45 min and is also an anti-malarial drug.

**Figure 9:**
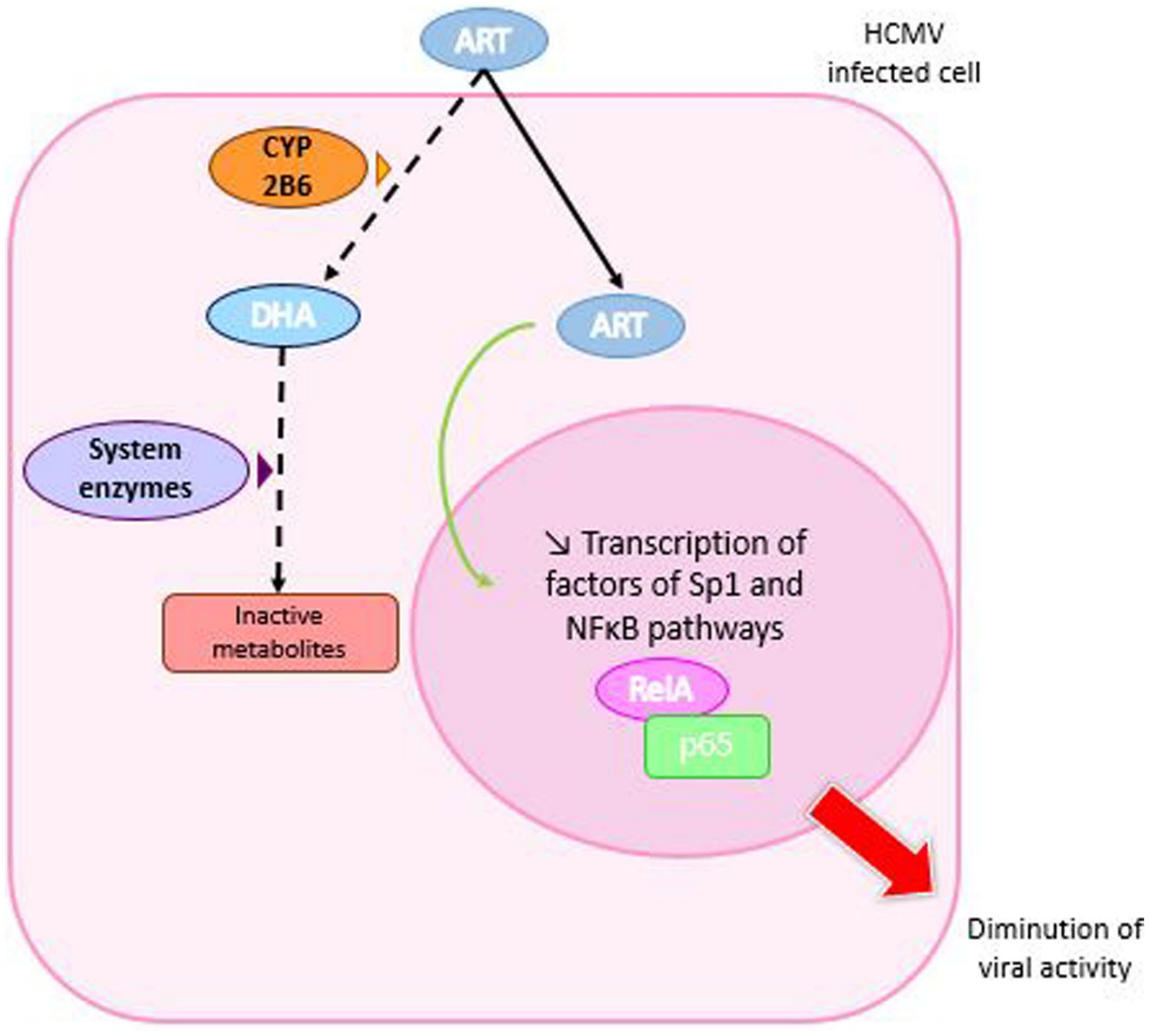
Mechanism of action of artemisinin derivatives. Artemisinin derivatives enter HCMV-infected cells and are metabolized to dihydroartemisin (DHA) by the cytochrome P-450 monooxygenase 2B6 (CYP 2B6) enzyme, which is then converted to inactive metabolites. In addition, ART moves to the cell nucleus where it inhibits the production of RelA and p65 involved in the NFκ-B pathway, which is necessary for viral replication.

In HCMV infections, ART inhibits cellular transcription factors Sp1 and NF-κB (Hutterer et al., 2015). Indeed, its derivatives, TF79 and TF27, inhibit NF-κB signaling by approximatively 90% at concentration of 3 μM to 0.3 μM, with a correlation between the diminution of NF-κB pathway and the antiviral activity (Hahn et al., 2018). Previous studies have suggested that sustained NF-κB activation is necessary for viral replication (Hiscott et al., 2001; Figure 9).

#### 4.1.2 Preclinical tests

Artemisinin derivatives are well tolerated in humans (Ribeiro and Olliaro, 1998; Adjuik et al., 2004). Rarely, slight and reversible adverse effects were observed such as first-degree heart block and neutropenia (Ribeiro and Olliaro, 1998; Adjuik et al., 2004). However, in animal models like mice, rats and dogs, neurotoxic effects were reported (Toovey, 2006). Studies showed that a shorter exposure to artemisinin derivatives with higher concentrations was less neurotoxic than a longer exposure with lower concentrations (Li et al., 2002).

ART was shown to be efficient against all types of herpesviruses, more than artemisinin. ART inhibits HCMV *in vitro* and *in vivo* (Efferth et al., 2002; Kaptein et al., 2006), both HHV-6 variants (Milbradt et al., 2009; Hakacova et al., 2013), Epstein–Barr virus (Auerochs et al., 2011) and Herpes simplex virus 1 (Efferth et al., 2008).

In addition, ART can be combined with other conventional anti-CMV drugs such as GCV, CDV and FOS to decrease risk of resistance mutation emergence. ART also shows a pronounced synergistic effect with MBV (Chou et al., 2011). Furthermore, its activity was confirmed in a model of 1^st^ trimester placental villi infection (Morère et al., 2015). In combination with MBV, ART showed a synergistic effect at low concentrations, with IC_50_ of 0.25 and 2 μM for the two molecules, respectively. However, the combination with baicalein had an antagonistic effect (Morère et al., 2015). In the same year, Drouot et al. (2016) demonstrated that combining ART with GCV, CDV and MBV was associated with synergy, while combining it with FOS or LMV produced only moderate synergy.

Artemisin was also tested against HCMV in different ratios in combination with the anti-HCMV drugs BDCRB, LMV, GCV, CDV, BCV and MBV. This study revealed synergistic antiviral activity with no microscopically apparent cell toxicity or reduction in cell viability (Oiknine-Djian et al., 2019).

ART derivative, TF27, also showed an anti-HCMV activity at nanomolar concentrations (Hutterer et al., 2015; Reiter et al., 2015; Fröhlich et al., 2018). Besides, antiviral activity against HCMV infections was also shown in *ex vivo* placental villi explant model (Jacquet et al., 2020). Recently, Sonntag et al., demonstrated its antiviral efficacy *in vivo* by using an established model of murine CMV infection of an immunodeficient mouse strain Rag−/− (Sonntag et al., 2019). TF27 has a higher antiviral activity than ART: the EC_50_ of 0.04 ± 0.01 μM against HCMV strain (Hutterer et al., 2015) was 100 fold lower than the EC50 of artesunate (Hahn et al., 2018).

#### 4.1.3 Clinical tests

ART is a good inhibitor for clinical use in the treatment of drug-resistant HCMV infection. The first report of treatment of CMV infection with ART in a HCT recipient with resistance to foscarnet and ganciclovir (DNA polymerase L776M mutation) dates back to 2008. Treatment with GCV, CDV and intravenous immunoglobulin had failed. ART was started at a dose of 100 mg daily after other treatments had been discontinued. A favorable response was observed, followed by a rapid reduction in viral load and improvement in hematopoiesis. During the 30 days of treatment, there were no adverse events and no increase in viremia. The patient received a third transplant with a recurrent episode of viremia but controlled by a new antiretroviral treatment regimen. Nevertheless, retinitis was diagnosed during treatment, reflecting the limited penetration of ART into the eye. Combining ART with GCV resolved this local infection and controlled the viral load. This study demonstrates the potential of ART to control CMV infection (Shapira et al., 2008).

ART was used in a case series of 6 SCT recipients as a preventive treatment for CMV infection to calculate its antiviral efficacy by studying viral kinetics. Two patients showed a decrease in viral load (0.8 to 2.1 log after 7 days). Antiviral efficacy was described as heterogeneous, ranging from 43 to 90%, and depended on the basic growth dynamics of the virus (Wolf et al., 2011).

In another study, ART was evaluated in five patients with resistant CMV infection. ART was unsuccessful in two cases of severe CMV disease with high CMV viral load and pulmonary involvement. However, these patients also suffered from diseases (Wegener’s granulomatosis and Hodgkin’s lymphoma) that may have accounted for some of the deaths. In conclusion, ART may be useful in the treatment of mild CMV disease due to multidrug-resistant strains. However, further data are needed on the risk factors associated with ART failure. In addition, it should be noted that ART is not sufficient to treat serious CMV disease with pulmonary involvement due to its poor diffusion in lung tissue, as has been reported in animal models (Zhao and Song, 1993; Germi et al., 2014).

#### 4.1.4 Resistance

To date, no resistance to artemisinin derivatives has been reported in CMV.

### 4.2 Flavonoids

Flavonoids are metabolites found in fruits and vegetables with high biological activity and low toxicity. Flavonoids with well-classified structures and well-defined structure–function relationships include flavans, flavanones, flavones, flavanonols, flavonols, catechins, anthocyanidins, isoflavones and chalcons (Tsao, 2010). Over 5,000 flavonoids were defined as molecules with potential health benefits as antioxidative, anti-inflammatory, antitumoral, antiviral and antibacterial effects (Middleton, 1998; Beecher, 2003; Cazarolli et al., 2008). Besides, flavonoids can specially modulate activities of cellular enzymes and inhibit protein kinases (Middleton, 1998). These metabolites are therefore a real option for current antiviral therapies. Indeed, it was shown that kaempferol inhibits herpes simplex virus (Amoros et al., 1992) and that baicalein and genistein interact with the first steps of HCMV infection (Evers et al., 2005). In an exploratory *in vitro* study, Cotin et al. (2012) showed that baicalein and quercetin were the most potent flavonoids to inhibit HCMV *in vitro*. Their combination had an additive effect. In addition, the combination of these two molecules with chalcone to reduce toxicity was tested against HCMV. The result was a synergistic effect for baicalein, while an antagonistic effect was observed with quercetin (Andouard et al., 2016). Both molecules were also combined with MBV, and quercetin did not improve the efficacy of MBV alone, unlike baicalein, which reduced infection by 90% at low concentrations (2.2 μM baicalein; 1 μM MBV; Morère et al., 2015). In this review, quercetin and baicalein will be explored for their potential antiviral activity.

#### 4.2.1 Quercetin

Quercetin or (3,3′,4′,5,7-pentahydroxy-2-phenylchromen-4-one; Figure 2) is the main representative of the flavonoid subclass, flavonols. The fruits and vegetables with the highest concentration of quercetin are apples, cherries, onions, asparagus and red leaf lettuce (Nishimuro et al., 2015). It is also found in herbs such as licorice.

In food, quercetin is present as quercetin glycosides that are hydrolyzed and released as aglycone. Then, aglycone is absorbed and metabolized into glucuronidated, methylated and sulfated derives (Kawabata et al., 2015). However, the stability of quercetin and its derivatives in the organism can be influenced by pH, temperature, metal ions and other compound as glutathione (GSH) (Boots et al., 2005; Moon et al., 2008). This could affect the efficacy of the molecule.

##### 4.2.1.1 Mechanism of action

**Figure 10:**
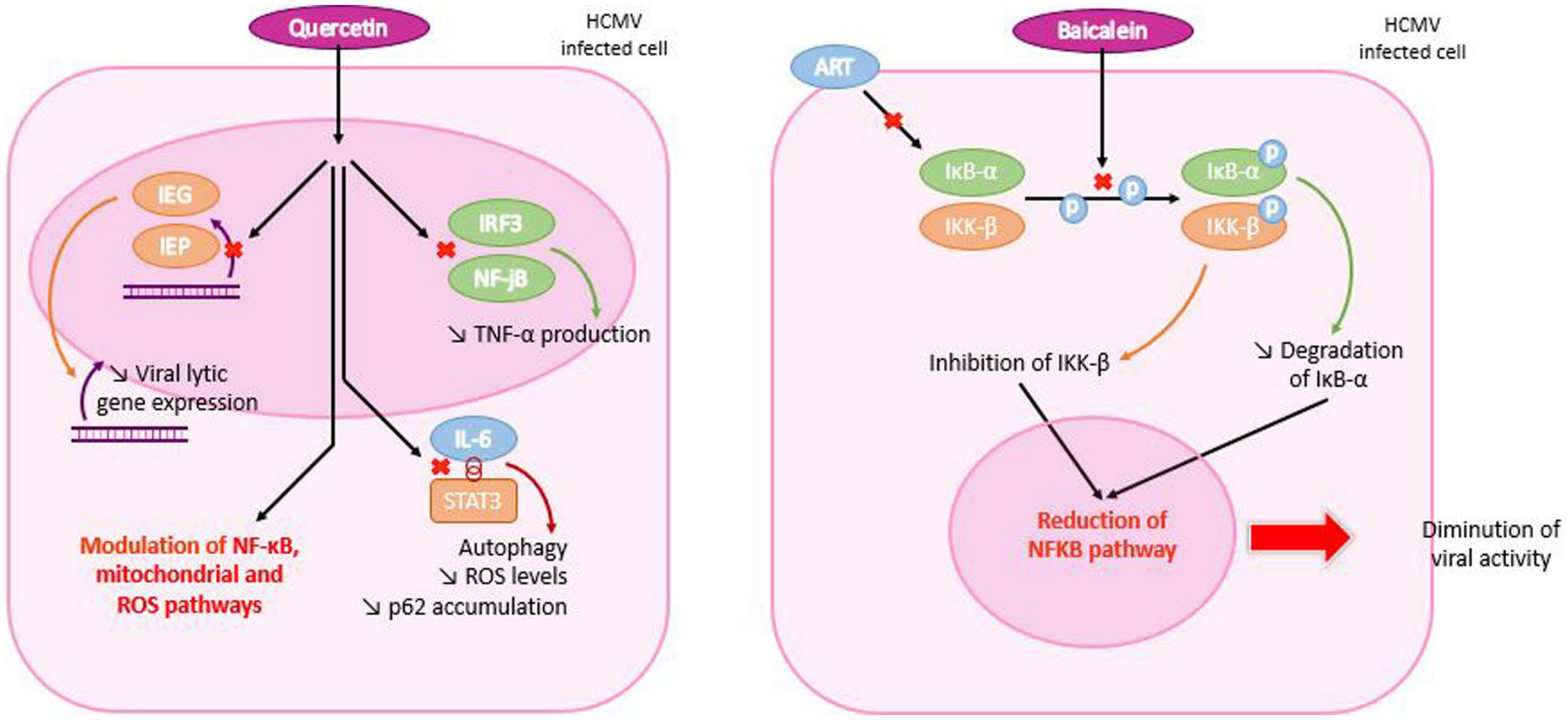
Mechanism of action of quercetin and baicalein in HCMV infected cells. Quercetin enters the HCMV-infected cell and travels to the nucleus, where it inhibits early viral protein and TNF-α production. It also inhibits contact between interleukin 6 (IL-6) and STAT3, resulting in reduced ROS levels and p62 accumulation. Both mechanisms are responsible for modulating NF-κB, mitochondrial and ROS pathways. In the case of baicalein, the molecule penetrates the cell and inhibits phosphorylation of IκB-α and IKK-β, which are degraded and inhibited, respectively. Each molecular mechanism leads to a reduction in the NF-κB pathway and, consequently, in viral activity.

Quercetin was shown to act by inhibition of early viral proteins IE-1and IE-2 expression (Cotin et al., 2012). Through the downregulation of IE-2 of VZV and HCMV, it inhibits viral lytic gene expression and replication (Kim et al., 2020) and modulates NF-κB, mitochondrial and ROS pathways (Hung et al., 2015; De Oliveira et al., 2016). Quercetin also inhibits the activation of IRF3 and NF-kB induced by HSV-1 infection in a TLR3-dependent manner that results in a lower production of TNF-α (Lee et al., 2017). Quercetin was then shown to prevent EBV-induced B cell immortalization and proliferation of lymphoblastoid cell lines by interrupting the dialectic between IL-6 and STAT3, promoting autophagy and reducing ROS levels and p62 accumulation (Granato et al., 2019; Figure 10).

##### 4.2.1.2 Preclinical tests

Many studies showed the antiviral activity of quercetin. Indeed, quercetin inhibits HBsAg and HBeAg secretion in Hepatitis B virus infected cells (Wu et al., 2007). It is also an active agent against HIV-1 reverse transcriptase, protease and α-glucosidase with an EC_50_ value of 60 μM (Yu et al., 2007).

Quercetin was tested *in vitro* against herpesvirus, reducing intracellular replication of HSV-1 and HCMV. The antiviral activity against HCMV infected cells was 4.8 μM and 145 μM against HSV-1 (Cotin et al., 2012). In 2022, a formulation of polyxamer 188 and quercetin (QP188) amplified the *in vitro* GCV antiviral activity against HCMV. Indeed, QP188 was tunable, bioactive and rapidly internalized in NIH/3 T3 cells. This formulation had a dose-dependent activity combined synergistically with GCV. These results could be interesting for finding means to reduce GCV toxicities (Kjar et al., 2022).

##### 4.2.1.3 Clinical study

In 2018, an herbal treatment (Gene-Eden-VIR/Novirin) composed of five ingredients including quercetin was tested in a clinical trial for the treatment of oral herpes. The study included 68 participants who took 1 to 4 capsules a day for an average duration of 10.4 months. Efficacy was assessed from symptom onset to complete resolution and also included analysis of recurrence rates. Treatment was compared with two conventional drugs: valaciclovir (VAVC) and aciclovir (ACV). Gene-Eden-VIR/Novirin was more effective in reducing the number and duration of oral herpes epidemics, and more secure than ACV and VAVC. Gene-Eden-VIR/Novirin reduced the duration of outbreaks from 5.83 days to 3.21 days in the treated group (*p* < 0.0001). In addition, 46.4% of patients on herbal treatment were relapse-free (*p* < 0.0001), and no adverse events were observed (Polansky et al., 2018). However, results of this study must be confirmed with further investigations. The same comparison was done with famciclovir (FCV) in 2016 (Polansky et al., 2016).

##### 4.2.1.4 Resistance

Currently, no resistance mutation to quercetin was documented.

#### 4.2.2 Baicalein

Baicalein (5,6,7-trihydroxyflavone; C15H10O5) (Figure 2) belongs to the flavone sub-family of flavonoids. This molecule is isolated from the roots of *Scutellaria baicalensis* with different properties: antioxidant, anti-inflammatory, anticancer, antidiabetic, antithrombotic, anxiolytic, anti-convulsive, cardioprotective, hepatoprotective and neuroprotective agent (Chang et al., 2016; Gao et al., 2016; Dinda et al., 2017; Cristelli et al., 2019; Tuli et al., 2020).

##### 4.2.2.1 Mechanism of action

Pretreatment with baicalein failed in suppressing viral replication in cells while post-treatment was effective. These results suggest that baicalein may be effective at the post-entry stage of viral infection (Luo et al., 2020). Previous studies indicated that baicalein had an inhibitory effect on NF-κB activation induced by pathological factors (Li et al., 2016).

*In vitro*, baicalein has been shown to act on IκB-α and therefore to have an antagonistic effect with ART, as they share the same target, which could lead to competitive inhibition (Morère et al., 2015). In agreement, Luo et al., reported that baicalein blocks NF-κB activation by inhibiting phosphorylation of IKK-β and IκB-α. By reducing IκB-α degradation, baicalein could inhibit viral replication (Luo et al., 2020). Thus, HSV-1 infections are prevented by a dual mechanism: the suppression of IKK-β phosphorylation and the decrease of NF-κB activation. This study also demonstrated that baicalein inactivates HSV-1 particles in a direct manner (Luo et al., 2020). However, further studies are needed to explain how baicalein acts on IKK-β phosphorylation (Figure 10).

##### 4.2.2.2 Preclinical tests

Baicalein has a poor oral bioavailability and a low aqueous solubility, which are the major disadvantage of this molecule. Studies of oral administration of baicalein have demonstrated that it is glucuronized in the intestinal wall and livers of rats and humans (Nagashima et al., 2000; Zhang et al., 2005, 2007). Additionally, baicalein is well absorbed by the small intestine and stomach (Taiming and Xuehua, 2006).

Some studies demonstrated baicalein is metabolized to baicalein and baicalein 6-O-sulfate in blood (Muto et al., 1998; Zhang et al., 2004; Dou et al., 2011). Following intravenous administration in rats, 75.7% of circulating baicalein in blood was as conjugated metabolites form (Lai et al., 2003). The bioavailability of baicalein in monkeys reached 23.0% after oral and intravenous administrations (Tian et al., 2012).

Cotin et al. (2012) demonstrated that baicalein inhibits *in vitro* CMV early proteins production. The inhibition of the tyrosine kinase activity of the EGF factor was already proved in a previous study (Evers et al., 2005). Additionally, combinations of quercetins and baicalein revealed additive effects particularly when baicalein was added at fixed quercetin concentrations. That reflected the probably higher efficacy of baicalein (Cotin et al., 2012).

##### 4.2.2.3 Clinical study

A randomized, double-blind, single-dose phase I trial of baicalein (100–2,800 mg) was conducted in 72 healthy adults. Analysis of baicalein and baicalin (baicalein’s 1st metabolite) was performed by liquid chromatography–tandem mass spectrometry on various biological fluids. Urinary clearance of baicalein and baicalin was 1, and 27% of baicalein was eliminated unchanged in feces. Eleven treatment-related side effects were recorded, but these were defined as moderated and resolved without further treatment. Baicalein is well tolerated by healthy patients, and no liver or kidney toxicity was observed (Li et al., 2014).

In 2021, another trial was conducted by Li et al. using data from the 2014 Phase I clinical trial. It was a randomized, placebo-controlled, multi-dose, and escalating trial of 36 healthy subjects who received 200, 400, and 600 mg of baicalein or placebo tablets. The drug was administered once on days 1 and 10, and three times daily from day 4 to 9. To analyze the pharmacokinetics of baicalein, blood and urine samples were taken from the 600 mg group. This study showed that baicalein tablets were safe and well tolerated. Mild adverse effects were observed, but none were not resolved. Maximum plasma concentrations were observed within 2 h of baicalein administration. Urinary excretion of baicalein and its metabolites peaked in 2 h, followed by a tendency to double the peak in 12 h. These results support the launch of a Phase II clinical trial (Li et al., 2021).

##### 4.2.2.4 Resistances

No resistance mutation to baicalein was reported in CMV.

### 4.3 Anti-COX-2

COX-2 inhibitors (cyclooxygenase-2) have been developed to reduce the adverse effects associated with the use of aspirin and indomethacin (gastric hemorrhage or perforation and hepatotoxicity; Nagi et al., 2015). Structural studies have highlighted the inhibitory activity of COX-2 as a heterocyclic or carbocyclic structure and substituting sulfonamide or methylsulfonyl in position para on one of the aromatic rings (Chakraborti et al., 2010). Nevertheless, some anti-COX-2 agents have been shown to cause serious adverse effects and have been withdrawn from the market (Mukherjee, 2002). Thus, certain anti-COX-2 agents have been identified in plants with fewer side effects than polyphenols. In this category, chalcones are a sub-category of the flavonoid family (Cerella et al., 2010).

#### 4.3.1 Mechanism of action

HCMV has been shown to increase the amount of COX-2 enzyme in infected cells, establishing an inflammatory state to promote replication (Speir et al., 1998). As part of the analysis of anti-inflammatory activity, certain chalcone derivatives and those of 2′-hydroxychalocone were determined to inhibit COX-2 and the production of PGE2 catalyzed by this enzyme. Chalcones appear to act before the early stage of viral replication, by reducing the production of IE-1 and IE-2 proteins (Andouard et al., 2021).

#### 4.3.2 Preclinical study

Celecoxib, a COX-2 inhibitor, was evaluated against HCMV in *in vitro* and *in vivo* (mouse) models of medulloblastoma. Its efficacy was compared with that of VGC. Both drugs inhibited HCMV replication *in vitro*, inhibited PGE2 production and reduced growth of medulloblastoma tumor cell *in vitro* and *in vivo* (Baryawno et al., 2011).

In a study carried out in 2021, the anti-HCMV activity of new 2′-hydroxychalcone compounds was assessed. These molecules were designed to inhibit PGE2 synthesis. To achieve this, a COX-2 pharmacophore (sulfonamide motif) and other substituents (chlorine, fluorine and methyl group) were introduced into the 2′-hydroxychalcone backbone (Zarghi and Arfaei, 2011). The selection of 4 anti-COX-2 agents was based on their significant activity against PGE2 production. However, three of them proved to be toxic to cells and one had a CC50 of 1,500 μM in growing cells and 185 μM in static cells, which was related to indomethacin (a non-specific cyclooxygenase inhibitor). Toxicity was potentially increased by the presence of the SO_2_NH_2_ group in the molecules, whereas the presence of the chlorine atom reduced it. These 2’hydroxychalcones were defined as less toxic than the tri-hydroxychalcones (Cotin et al., 2012). The molecules were tested against strain AD169-GFP. EC50s were up to 16 times higher than GCV (EC50 = 19.6 ± 10.1 μM; 8.6 ± 10.1 μM; 10.5 ± 3.6 μM; 22.1 ± 7.7 μM; 15.1 ± 5.9 μM for the 4 chalcones and indomethacin respectively). EC50s were also determined on clinical isolates and proved effective against resistant strains. Three chalcones tested proved capable of inhibiting IE1-72 production (Andouard et al., 2021).

In addition, a synthesized anti-COX-2 was combined with other anti-CMV drugs such as GCV, MBV, baicalein, quercetin and ART. This resulted in a synergistic effect with MBV or baicalein. An additive effect was demonstrated with GCV or ART, and an antagonistic effect was observed with quercetin (Andouard et al., 2021).

#### 4.3.3 Clinical study

No clinical study was done for anti-COX-2 molecules against HCMV (source: ClinicaTrials.gov).

#### 4.3.4 Resistances

So far, no resistance mutations in HCMV genome were defined.

## 5 Immunomodulating molecules

### 5.1 Leflunomide

Leflunomide (LEF) (HWA 486; A77 1726, Arava®) or N-(4-trifluoromethylphenyl)-5-methylisoxazol-4-carboxamide (Figure 2) is an antirheumatic agent used to treat rheumatoid arthritis. It was also demonstrated as effective in HCMV infection in HCT and renal transplant.

#### 5.1.1 Mechanism of action

**Figure 11:**
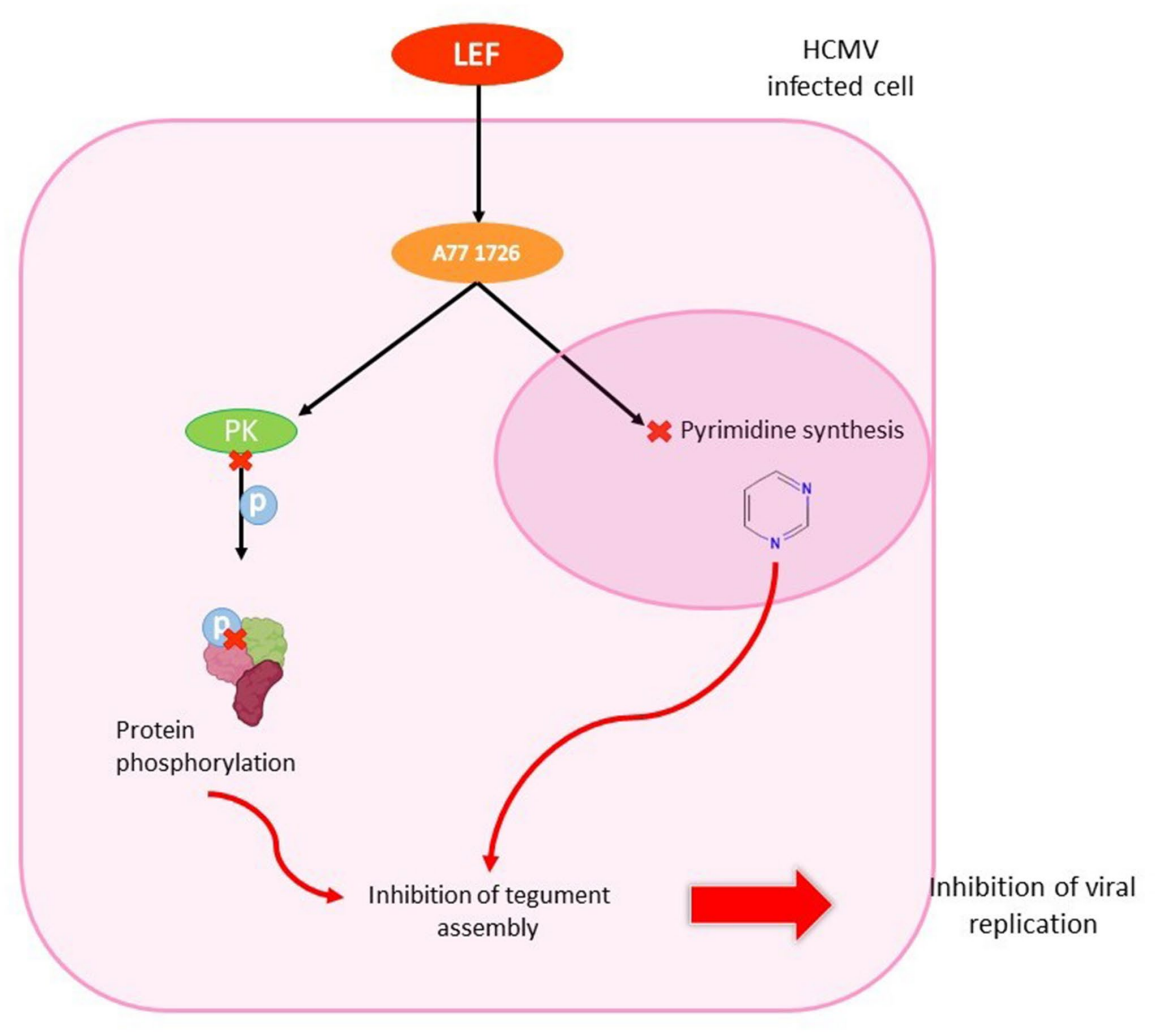
Antiviral effect of leflunomide in HCMV infected cell. LEF enters the HCMV-infected cell and is converted to teriflunomide (A77 1726). The metabolite inhibits protein kinase, which is unable to phosphorylate proteins. It also penetrates the nucleus to inhibit pyrimidine synthesis. These two mechanisms are involved in inhibiting tegument assembly, thereby stopping viral replication.

After administration, LEF is converted to an active matabolite, teriflunomide (A77 1726) that blocks lymphocyte enzyme dihydroorotate dehydrogenase and the pyrimidine biosynthetic pathway (Williamson et al., 1996). This activity results in a lower T-cell proliferation and changes in immune response (Dayer and Cutolo, 2005; Chong et al., 2006). At a later stage in virion assembly, it prevents viral nucleocapsides from acquiring integument. LEF has a dose-dependent effect on the infectious production of HCMV (Waldman et al., 1999a). Unlike polymerase inhibitors, LEF did not inhibit HCMV DNA replication, but it did appear to interfere with tegument assembly by inhibiting protein phosphorylation (Xu et al., 1995; Waldman et al., 1999b; Figure 11).

##### 5.1.1.1 Preclinical tests

HCMV isolates in human fibroblats and endothelial cells, including multidrug-resistant viruses (EC_50_ 40–60 μM), are inhibited by LEF (Waldman et al., 1999b). LEF also inhibits HSV-1 with same mechanism of action than with HCMV (Knight et al., 2001).

Furthermore, the LEF was evaluated *in vivo* using animal models. Immunodeficient rats were inoculated with rat CMV (Maastricht strain of RCMV) and administered 15 mg/kg/day LEF for 14 days, 10 mg/kg/day GCV for 5 days, or a drug-free vehicle. Plaque assay from tissue homogenates (salivary glands, spleen and lung) showed a decrease of 75 to 99% of viral load in the organs of animals treated with LEF, and 85 to 99% in those treated with GCV. Thus, LEF is an effective agent in decreasing viral load *in vivo* (Waldman et al., 1999a). After that, it was demonstrated the efficacy of LEF in an allogenic cardiac transplant model of RCMV infection with low toxicity (Chong et al., 2006).

##### 5.1.1.2 Clinical study

Due to its synergy with calcineurin phosphatase inhibitors and its inhibitory effects on herpesvirus replication, LEF was presented as a promising drug for experimental transplantation (Williams et al., 2002). Fifty-three recipients of LEF were analyzed in a retrospective study. A single-dose pharmacokinetic study was performed in stable renal transplant recipients with a target serum concentration of 100 μg/mL to require a loading dose of 1,200 to 1,400 mg over a 7-day period. Anemia in the renal transplant patients and increase of liver enzymes in liver-transplanted patients were the major observed toxicities (Williams et al., 2002). Another study reported same side effects and diarrhea after a therapy with a mean duration of 3.5 months. The recommended dose of LEF was 100 mg/day for 5 days followed by 40 mg/day, based on the serum metabolite levels of A77 1726 teriflunomide (Avery et al., 2010).

In one study, John and colleagues analyzed 17 patients who underwent kidney transplantation and were infected with CMV. Patients were treated with monitored doses of leflunomide. Among these 17 patients, 88% responded clinically to leflunomide therapy with viral clearance in the blood and healing of the organs involved. The cost of treatment was cheaper than that of ganciclovir: 64 $ for 6 months against 721 $ for 2 weeks, respectively, (John et al., 2005).

Three cases of resistant HCMV infections were reported with LEF treatment. It was showed that LEF is not efficient enough in monotherapy and should be combined with GCV or FOS to better control CMV infection. It was also only used for CMV maintenance therapy (El Chaer et al., 2016). As an oral treatment, LEF is also a convenient alternative that does not need to stay in hospital to reach undetectable viral load (Gómez Valbuena et al., 2016). Then, after lung transplant, LEF was assessed in a case of drug resistant CMV retinitis. In spite of intravitreal FOS administration and oral VACV, HCMV disease progressed. Oral LEF helped in control of retinitis and allowed cessation of intravitreal treatment. No recurrence of infection was noticed (Rifkin et al., 2017).

A more recent study on case series assessed LEF in patients treated with GCV and FOS with adverse effects reported in 50% of cases. In 66.67% of cases, resistance mutations to polymerase inhibitors were present before LEF treatment. LEF was prescribed to treat HCMV infection in 75% of patients and as secondary prophylaxis in 25% of them. A primary reduction of HCMV viremia was observed after the beginning of LEF treatment in 77.7% of recipients but was transient in 22.2%. In 58.3% of recipients, LEF suppressed HCMV infection for long-term. Adverse effects were responsible for treatment discontinuation in 25% of cases. This study showed that LEF can be an effective treatment for transplant recipients with GCV-resistant infections, whether alone or combined with other drugs, even though the small number of subjects was a limitation. It can also be used as a secondary prophylaxis (Silva et al., 2018). LEF was also proposed in combination with hyperimmune globulins in cardiothoracic grafts and was associated with decreasing viremia (Santhanakrishnan et al., 2019).

In other hand, there is few numbers of studies with LEF in allogeneic HCT. One reported that LEF had efficacy in HCMV clearance in 38% of cases. Nevertheless, treatment significantly succeeded (53%; *p* = 0.022) only when LEF was used in patients with HCMV viral load <2.10^3^copies/mL. Furthermore, it was demonstrated as ineffective in patients with terminal organ disease. Thus, LEF could be used in prophylaxis in stem cell transplants (Gokarn et al., 2019).

##### 5.1.1.3 Resistance

Currently, there is no reported resistance mutation to leflunomide in HCMV.

### 5.2 mTOR inhibitor: everolimus

Everolimus (SDZ RAD; Certican®, ZORTRESS®; EVR) or 40-O-(2-hydroxyethyl)-rapamycin (Figure 2) is an oral mammalian target of a sirolimus-derived rapamycin inhibitor. It is used in immunosuppressive therapy after SOT. FDA approved it for the prevention of rejection in kidney transplant recipients at low to moderate risk (Gabardi and Baroletti, 2010).

#### 5.2.1 Mechanism of action

**Figure 12:**
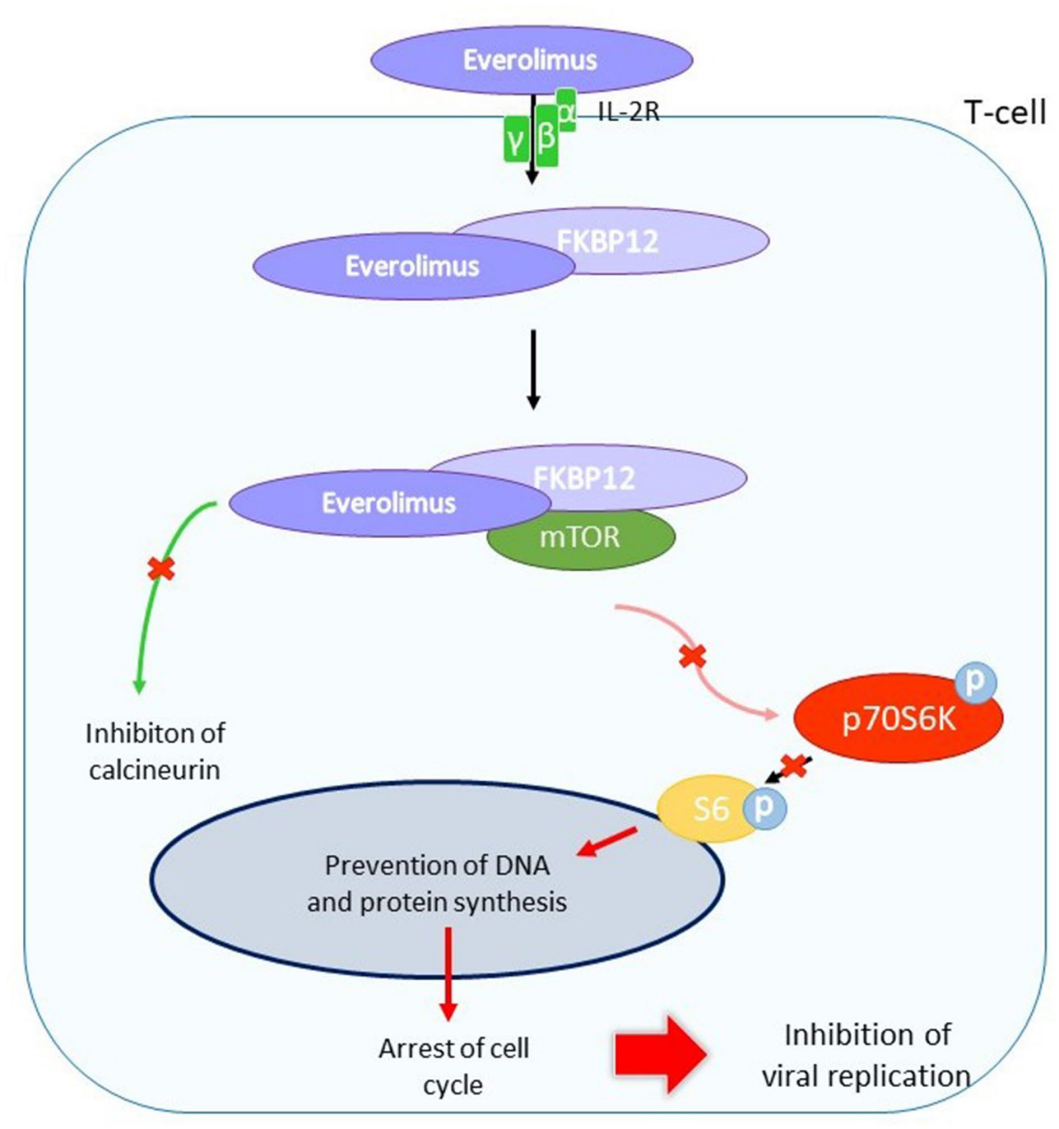
Indirect antiviral action of everolimus in HCMV infected T-cell. EVR binds to the interleukin-2 receptor (IL-2R) on HCMV-infected T cells. The molecule enters the cell and binds to the immunophilin FKBP12. The complex binds to mTOR, which is unable to phosphorylate the p70S6 kinase. The kinase does not phosphorylate S6, which is involved in DNA and protein synthesis. HCMV replication is inhibited by cell cycle arrest.

Intracellular immunophilin (FKBP12) is bound by EVR, but it does not inhibit calcineurin, it binds to the mechanistic target of Rapamycin (mTOR). It is at the origin of the inhibition of a multifunctional serine–threonine kinase, preventing both the synthesis of DNA and proteins that leads to a cell cycle shutdown. More precisely, after stimulation of the IL-2 receptor on the activated T-cell, EVR inhibits p70S6 kinase which acts at a later stage in the T-cell mediated response (Roy and Arav-Boger, 2014). EVR inhibits HCMV through improved CD8+/CD4+ T cell responses specific to HCMV (Havenith et al., 2013; Roy and Arav-Boger, 2014; Figure 12).

##### 5.2.1.1 Preclinical tests

So far, no preclinical test of the *in vitro* anti-HCMV efficacy of EVR was done because it is not the primary function of this molecule.

##### 5.2.1.2 Clinical study

The effectiveness of EVR against HCMV has been demonstrated for several years. A multicenter Phase III trial included 634 heart transplant patients receiving immunosuppressive therapies (1.5 mg/day EVR; 3 mg/day EVR or azathioprine). The EVR groups had significant lower HCMV incidence (*p* < 0.001). Thus, the use of EVR suggests an additional benefit in cardiac transplant recipients as it is linked to lower rates of HCMV infection, syndrome or organ damage (Hill et al., 2007). In accordance with these results, same doses of EVR were assessed in heart and renal transplants recipients compared to mycophenolic acid (MPA). A significantly lower HCMV incidence was observed in the EVR group at 3.0 mg (3%) than in the MPA group (7%) (*p* < 0.04). The same observation was made for organ involvement in the EVR 1.5 mg group (0.9%) than in the MPA group (3%) (p < 0.04). Therefore, the EVR was associated with a decrease in EVR events compared to the MPA (Brennan et al., 2011). In a study of data from 3 randomized trials of *de novo* cardiac transplant recipients, Kobashigawa et al. reported that EVR was linked to a significantly lower incidence of HCMV infections compared to azathioprine and mycophenolate mofetil by combining its immunosuppressive efficacy with antiproliferative effects that may have a positive impact on long-term results (Kobashigawa et al., 2013).

In another study, EVR was compared with valganciclovir (VGC) in heart transplant recipients. EVR was introduced at 1.5 mg/day to discontinue the use of mycophenolate mophetil in combination with VGC that caused neutropenia. HCMV antigenemia was negative even after discontinuation of VGC and both renal function and neutrophil counts were normalized. No major side effects or rejection due to EVR were observed. Thus, EVR was described as an alternative or additive option in immunosuppressive therapy for heart transplant recipients because of maintenance of immune tolerance, prophylactic potency against HCMV, reduced myelosuppression and potential sparing of renal function (Imamura et al., 2012).

The impact of EVR on HCMV infection at both systemic or pulmonary level was also evaluated in lung transplant recipients (*n* = 32 patients). Eighteen patients were on EVR-immunosuppressive regimens. No differences were described in HCMV viremia occurrence between EVR-based and EVR-free immunosuppressive regimens. However, patients with EVR treatment experienced fewer high-load HCMV episodes defined as ≥10^5^ copies/mL during EVR administration. It validate the reduction of HCMV events in EVR-based regimens transplanted patients, as lung transplant recipients (Rittà et al., 2015).

The international randomized phase IV TRANSFORM trial (NCT01950819) was conducted in *de novo* renal transplant patients randomized to RVE with reduced exposure to CNI or MPA with standard exposure to CNI, treated with induction and corticosteroids. EVR caused more adverse reactions than MPA, such as hyperlipidemia, interstitial lung disease, peripheral edema, proteinuria, stomatitis/mouth ulceration, thrombocytopenia and wound healing complications. However, EVR has been associated with viral infections less frequently than MPA. Indeed, HCMV infections and HCMV syndrome were lower (8.1% vs. 20.1%; *p* < 0.001 and 13.6% vs. 23.0%; *p* < 0.044 respectively). The same result was observed for BKV infections. EVR was more often stopped due to rejection or delayed healing (Tedesco-Silva et al., 2019). Another phase IV trial (NCT02096107) with EVR in kidney transplants was also conducted and reported that EVR with low tacrolimus exposure produced related efficacy to tacrolimus and MPA with significantly lower BK and HCMV levels (Taber et al., 2019). A more recent Phase IV trial was conducted with 186 seropositive HCMV kidney recipients randomized (1:1) to receive EVR or MPA in combination with basiliximab, cyclosporine and steroids. In seropositive recipients, HCMV DNAemia is prevented by EVR treatment until it is no longer tolerated or stopped (Kaminski et al., 2022a). In addition, the same authors reported that the T-cell phenotype may offer a new biomarker for predicting post-transplant infection and classifying patients who should be eligible for EVR treatment (Kaminski et al., 2022b).

##### 5.2.1.3 Resistances

Currently, there is no resistance mutation to EVR that was reported in CMV.

## 6 Immunoglobulins

Two types of immunoglobulins are available in therapy: intravenous administered immunoglobulins (IVIG) and hyperimmune immunoglobulins (CMV-HIG). We will focus on HCMV-specific immunoglobulins which are able to neutralize viral infectivity (Aiba et al., 2017). Immunoglobulins may be used in preventing congenital infections or in association with other antivirals to cure HCMV infection in transplantation (El-Qushayri et al., 2021).

CMV-HIG are obtained by purification of adult human plasma-derived immunoglobulin products in pools selected for high levels of anti-HCMV antibodies. IVIG are acquired from the plasmas of healthy blood donors on the basis of a high level of antibodies against HCMV (Maniez-Montreuil et al., 1984). Using a correlation test between HCMV antibody titer and viral neutralization titer, it was shown that HIG has a higher level of anti-HCMV IgG than IVIG (Germer et al., 2016; Schampera et al., 2017). Currently, there is two options of HCMV-HIG which are, respectively, authorized in United States and Europe: Cytogam® (CMVIG CG; CSL Behring, Berne, Switzerland) and Cytotect CP® (Biotest AG).

Cytogam® is an HCMV-HIG derived from human plasma with high titers of anti-HCMV antibodies [112,5 PEIU/mL (Paul Ehrlich Institute Units)] (Germer et al., 2016). It contains a standardized amount of Ig (5 ± 0.1%). In the United States, it is approved for the prophylaxis of HCMV disease in heart, liver, lung, kidney and pancreas transplant recipients.

Cytotect CP® contains 5% Ig (50 mg/mL), 96% of which is IgG (110.1 PEIU/mL) (Germer et al., 2016). Its maximum IgA level is 2 mg/mL, and its anti-HCMV antibody level is 100 U/mL. It is approved in Europe for the prophylaxis of HCMV infection in patients treated with immunosuppressants and in solid organ transplant patients. In France, this solution is available as part of a patient-nominated program for the prevention or treatment of HCMV infection.

Cytotect CP® and Cytogam® preparations have high avidity indexes (90.5 and 91.0% respectively) and both have been tested in immunoblot assays against antigenic HCMV glycoproteins with similar results (Germer et al., 2016).

### 6.1 Mechanism of action

**Figure 13:**
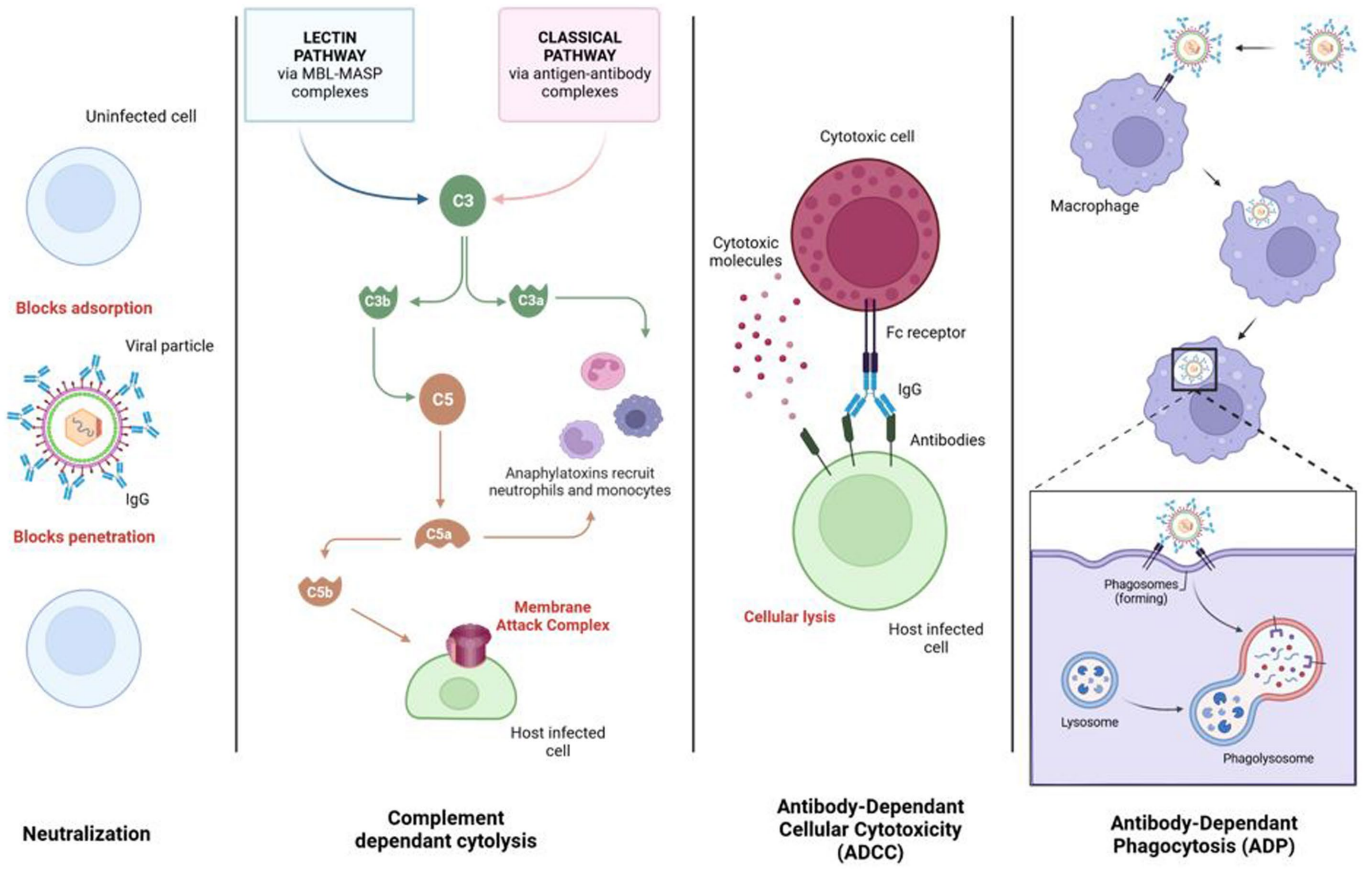
The four modes of action of anti-CMV immunoglobulins. In the first mechanism, HIGs neutralize the viral particle by binding its epitope to viral envelope glycoproteins. Virus adsorption and cellular penetration are inhibited. In the second mechanism, HIGs induce complement pathways leading to the formation of the membrane attack complex (MAC), which creates a hole in the cell membrane leading to cytolysis. In the third mechanism (ADCC), the Fc fraction of the HIG-neutralizing viral particle is recognized by the cytotoxic cell’s Fc receptor (FcR). This leads to the release of cytotoxic molecules that create a cytolysis signal. In the final mechanism, after neutralization of the viral particle by HIGs, macrophage FcRs recognize HIG Fc, resulting in a phagocytosis signal. The viral particle-HIG complex is phagocytosed. The phagosome fuses with the lysosome, forming a phagolysosome and destroying the complex. Created with BioRender.com

HCMV-HIG involve the neutralization of viral particles in extracellular environment, opsonization for phagocytosis (ADP), activation of the cellular immune system (ADCC) and immune adaptation with complement activation (Carbone, 2016; Figure 13).

### 6.2 Preclinical tests

Efficacy of HCMV-HIG was proved *in vitro* with a higher HCMV neutralizing activity than IVIG (Miescher et al., 2015; Germer et al., 2016; Schampera et al., 2017).

In 2022 and 2023, an *in vitro* and *ex vivo* (in placental villi of the first trimester) study analyzed the efficacy and mode of action of Cytotect CP® and showed good efficacy and low toxicity in different routes of HCMV infection (Coste Mazeau et al., 2022, 2023). The development of infection foci was blocked by Cytotect CP® with a DN50 of 0.011 to 0.033 U/mL on endothelial strains (TB40E/VHLE) in *in vitro* neutralization assays. Samely, on day 7, Cytotect CP® prevents CMV infection with an EC_50_ of 0.024 U/mL in placental villi. Although, once infected, viral growth was not inhibited in explants. The viability of villi has not been affected. An additional study shows the need for renewing Cytotect CP® every 7 days in the medium to maintain efficacy at day 14 in the explants. This was coherent with the recent pharmacokinetics study from Kagan et al. showing the decrease of plasma concentrations within 2 weeks (Kagan et al., 2021).

Potency of CMV-HIGs during pregnacy was assessed in animal models. Guinea pigs were used as model to study HCMV-HIG because congenital infection has similarities between HCMV and GPCMV. The placental barrier can be crossed by both viruses that can create an *in utero* infection. In guinea pig experiments, pup survival is the endpoint as GPCMV causes their death (Schleiss, 2008). This model evaluated passive immunization of the fetus. Indeed, studies have used pregnant guinea pigs with GPCMV infection prior to HIG administration or after to assess the inhibition of viral particle and gB. In two studies, fetal survival was increased after administration of CMV-HIG but the viral load was not affected (Bia et al., 1980; Chatterjee et al., 2001). Thus, anti-gB CMV-HIG were used in another study and allowed a reduction in fetal infection, inflmmation of placenta, death of the fetus and increased fetal development. Results were independent of CMV-HIG administration time. Infection rate of fetuses was significantly reduced with the administration of CMV-HIG from 39 to 0%. Anti-gB HIG also reduced inflammation of placenta and increased fetal development (Bratcher et al., 1995).

The mouse model was used to assess passive immunization in the fetal brain because the mouse CMV (mCMV) does not pass through the placental barrier. Peritoneal cavity of new-born mice was infected with mCMV. The viral infection was disseminated in mice brains with associated inflammatory lesions such as infiltrations of mononuclear cells and prominent glial nodules. Treatment with HIG or monoclonal antibody specific of gB glycoprotein led to decrease of viral load in the brains and a 5-fold reduction of inflammatory lesions. This study showed that CMV-HIG are responsible for a limitation of viral replication and its associated lesions in the brain (Cekinović et al., 2008).

### 6.3 Clinical trials

CMV-HIG treatments were shown to be effective against severe HCMV infections in immunocompromised patients and congenital infections. Whereas, retrospective studies assessed the potency of the HCMV-HIG for the prevention of cCMV infection. To date, the only two published trials have reported that anti-HCMV IgG has not been effective (Revello et al., 2014; Hughes et al., 2021). The effectiveness of treatment is determined according to the frequency of treatment, the concentration of HCMV-HIG and the time of seroconversion of the patient (Kagan et al., 2019, 2021). A new administration protocol with higher concentrations at the first stage of pregnancy will begin as a third Phase III trial (NCT 05170269).

Concerning adverse events, in the phase II study, Cytotect® CP was associated with a tendancy to a low birth weight for treated newborns (Revello et al., 2014). Nevertheless, these results were disclaimed by a newer investigation of Chiaie et al. that was conducted in 50 women with a dose of 200 unit/kg taken twice during pregnancy. No side effects caused by HCMV-HIG were reported in this study (Chiaie et al., 2018).

Recently, an observational study was undertaken in 149 pregnant women to assess the effectiveness of Cytotect CP® in pregnant women with HCMV primary infection during the first trimester or periconceptional period. This study was based on pharmacokinetics results showing the half-life of HCMV-HIGs, which is about 10 days. Every 2 weeks, a dose of 200 IU/kg body weight of Cytotect CP® was injected to women with primary infection before 14 weeks of amenorrhea. Intravenous injection should begin within 3 weeks of discovery of the primary infection. HCMV-HIG injections were made until the 18^th^ week of amenorrhea. A significantly lower rate of maternal infection transmitted to the fetus was observed with 7.5% in the intervention group and 35.2% in the control group (Kagan et al., 2021).

In comparison with the historical cohort of Feldman, Seidel et al. conducted an observational survey using the same doses as Kagan et al. and found a significant reduction in the rate of mother-to-fetus transmission, regardless of the term of pregnancy (23.9% versus 39.9% for the Feldman control group; *p* = 0.026; Feldman et al., 2011; Seidel et al., 2020).

Some observational studies suggest that HCMV-HIG may have a protecting effect on the fetus after maternal primary infection (Buxmann et al., 2012). Cytotect® CP was evaluated on 592 cases of maternal primary infection before the 19th week of amenorrhea. After an administration of 200 unit/kg, HCMV-linked symptoms in the newborn decreased (Visentin et al., 2012).

Nevertheless, Cytotect CP® has also been evaluated in rescue therapy for hematopoietic stem cell recipients with resistant refractory infections. One study determined the safety and efficacy profiles of Cytotect CP® in 23 patients with refractory HCMV and GVHD (74%) and/or steroid treatment (64%). After 15 days, a response was observed in 18 patients. Of the 18, four had CMV reactivation, one died of CMV infection, and 4 died of CMV-related causes within 100 days of starting treatment. Total 100-day survival was 69.6% after Cytotect CP®. However, no statistical difference between respondents and non-respondents was reported. Thus, it showed that Cytotect CP® was well tolerated and effective as a recovery treatment (Alsuliman et al., 2018).

Another rescue therapy study was conducted in cardiothoracic transplant recipients. This was a 6-year retrospective single-center experiment in 35 patients. The rescue therapy consisted of Cytotect CP® supplemented by antiviral treatment (GCV/VGCV/LEF). HCMV-HIGs were well tolerated by patients and safe; only two patients had adverse events, but their symptoms were resolved after reducing HCMV-HIG doses to 1.5 mg/kg. CMV DNA was reduced in all patients and, after 4 weeks, undetectable in 73% of them. HCMV-HIG were shown as effective to control viral replication in cardiothoracic transplant recipients (Santhanakrishnan et al., 2019).

### 6.4 Resistances

So far, there is no reported case of resistant CMV to HIG.

## 7 Discussion

The CMV DNA polymerase inhibitors (GCV, FOS, and CDV) are essential molecules for decreasing the morbidity and mortality rate associated with CMV infection in transplant recipients (SOT or HSCT). However, they are often responsible for toxicity (hematologic or renal) and emergence of resistance that may limit their use. Nevertheless, they are always the most used in clinical practice. The approval of letermovir represents an important innovation for CMV prevention in HSCT. A decisive step forward in the management of refractory and/or resistant infections has been achieved with the validation of maribavir in transplant recipients. However, in case of multidrug resistance, or in non-transplanted patient or also in the prevention and treatment of cCMV infection, finding new antivirals or molecules able to inhibit CMV replication with the lowest toxicity remains a critical need.

In this review, we have listed the main compounds with potential activity against CMV. They belong to different families of molecules, some with specific antiviral activity, others known for their antimicrobial or immunosuppressive activities but with anti-CMV efficacy, and still others with a completely different mode of action, such as immunoglobulins.

Some direct antivirals like brincidofovir or cyclopropavir have an interesting profile for CMV treatment, but the development of the first was stopped after emergence of toxicity in a phase II clinical trial whereas the second did not enter in phase II trials until now. The search of cidofovir derivatives or pro-drugs is not yet stopped: recently, a new family of antiviral acyclonucleoside analogs with high bioavailability and potential activity against HCMV was patented (Roy and Agrofoglio, 2022) and are under evaluation (personal data). Concerning anti-terminase benzimidazole analogs, their poor biodisponibility limited their clinical use. As the effectiveness of letermovir validates terminase inhibitors as a clinically relevant class of antiviral agents, the development of other terminase inhibitors may be considered and research on these inhibitors should be encouraged (Ligat et al., 2018; Gentry et al., 2019). Indirect antivirals are also an interesting area to explore because of their cellular targets, which do not select for resistance to direct antivirals. Artemisin derivatives have shown their efficacy to control HCMV replication in some transplant patients but with varying degrees of effectiveness. A degree of uncertainty therefore remains when these treatments are used as monotherapy but artesunate is still an alternative alone or in association in multidrug resistant infections. Other indirect antivirals like flavonoids or anti-cox derivatives have demonstrated good efficacy *in vitro* but few or no study were performed until now with these molecules. Expanding these families of chemical compounds could be a complementary approach. Concerning immunomodulating agents, leflunomide, mTOR inhibitors and immunoglobulins could be used in combination with other antiviral drugs, as their use as monotherapy is not sufficiently effective to be recommended for the control of high level HCMV replication.

With agents acting by new modes of action as LTV and MBV available in clinical use, association therapy for the treatment of CMV infection and disease can move from concept to reality. *In vitro* studies support at least one additive (and sometimes synergistic) effect of association of LTV or MBV with DNA polymerase inhibitors. MBV targeting the kinase pUL97, his mode of action differs from the DNA polymerase or terminase inhibitors, but pUL97 being the kinase essential for GCV activation, association of both antivirals are antagonist. Moreover, we and others have already demonstrated *in vitro* that combination of indirect antiviral with DNA polymerase inhibitors have additive or synergistic activity (Morère et al., 2015; Wildum et al., 2015; Drouot et al., 2016; Chou et al., 2019). So far, clinical studies are needed to assess which combination therapy for HCMV is superior to monotherapy and which combination regimens are most effective. Combination therapy has already proved its relevance to treat other viral infections such as human immunodeficiency virus and hepatitis C virus (Hepatitis, 2018; Saag et al., 2020). Use of immunoglobulins in addition with antiviral therapy should also be considered in immunosuppressed patients, especially those with weak or null cellular response against CMV. Nevertheless, the effectiveness of this approach must be confirmed in clinical trials to better define the indications according to the patient profile, the history of CMV infection and the antivirals already used. Since few agents are currently being studied in humans, a combination therapy with existing agents and possibly with indirect acting anti-HCMV molecules approved for other indications not suitable for use in monotherapy should be considered.

## Author contributions

CG: Writing – original draft. SA: Writing – review & editing. SH: Writing – review & editing.
